# Auxin regulates source-sink carbohydrate partitioning and reproductive organ development in rice

**DOI:** 10.1073/pnas.2121671119

**Published:** 2022-08-29

**Authors:** Zhigang Zhao, Chaolong Wang, Xiaowen Yu, Yunlu Tian, Wenxin Wang, Yunhui Zhang, Wenting Bai, Ning Yang, Tao Zhang, Hai Zheng, Qiming Wang, Jiayu Lu, Dekun Lei, Xiaodong He, Keyi Chen, Junwen Gao, Xi Liu, Shijia Liu, Ling Jiang, Haiyang Wang, Jianmin Wan

**Affiliations:** ^a^National Key Laboratory for Crop Genetics and Germplasm Enhancement, Nanjing Agricultural University, Nanjing 210095, China;; ^b^National Key Facility for Crop Gene Resources and Genetic Improvement, Institute of Crop Science, Chinese Academy of Agricultural Sciences, Beijing 100081, China;; ^c^National Key Laboratory of Crop Genetic Improvement and National Center of Plant Gene Research, Huazhong Agricultural University, Wuhan 430070, China

**Keywords:** auxin, source-sink, carbohydrate partitioning, reproductive organ, rice

## Abstract

Carbohydrate partitioning between the source and sink tissues plays an important role in regulating plant growth and development. However, the molecular mechanisms regulating this process remain poorly understood. In this study, we show that elevated auxin levels in the rice *dao* mutant cause increased accumulation of sucrose in the photosynthetic leaves but reduced sucrose content in the reproductive organs (particularly in the lodicules, anthers, and ovaries), leading to closed spikelets, indehiscent anthers, and parthenocarpic seeds. RNA sequencing analysis revealed that the expression of *AUXIN RESPONSE FACTOR 18* (*OsARF18*) and *OsARF2* is significantly up- and down-regulated, respectively, in the lodicule of *dao* mutant. Overexpression of *OsARF18* or knocking out of *OsARF2* phenocopies the *dao* mutant. We demonstrate that OsARF2 regulates the expression of *OsSUT1* through direct binding to the sugar-responsive elements (SuREs) in the *OsSUT1* promoter and that OsARF18 represses the expression of *OsARF2* and *OsSUT1* via direct binding to the auxin-responsive element (AuxRE) or SuRE in their promoters, respectively. Furthermore, overexpression of *OsSUT1* in the *dao* and *Osarf2* mutant backgrounds could largely rescue the spikelets’ opening and seed-setting defects. Collectively, our results reveal an auxin signaling cascade regulating source-sink carbohydrate partitioning and reproductive organ development in rice.

## Significance

Effective communication between source organs and sink organs is pivotal in carbohydrate assimilation and partitioning during plant growth and development. Auxin is required for many aspects of plant growth and development. However, very little is known about how these two important classes of molecules coordinate and co-regulate plant developmental processes. In this study, we elucidate an OsARF18-OsARF2-OsSUT1–mediated auxin signaling cascade regulating carbohydrate partitioning between the source and sink tissues in rice, which is essential for proper development of rice reproductive organs. Our findings represent a major step forward in increasing our knowledge of sucrose transport regulation in plants and have important implications in improving crop yield through better coordination of source and sink activities.

In higher plants, the sink organs, such as flowers, fruits, and seeds, are heterotrophic in nature and rely on nutrients supplied from the photosynthetically active organs (e.g., leaves, termed source organs) for their growth and development (1–4). Higher plants utilize the phloem sieve elements for long-distance transport of nutrients (mainly sucrose) from the source to the sink organs. The turgor pressure generated through the osmotic effect of sucrose loading into the phloem at the source and the unloading at the sink creates the driving force for long-distance translocation of all other compounds, including nutrients, water, and signaling molecules in the phloem (3, 5).

Sucrose translocation is mainly mediated by two sucrose transporter families: Sugars Will Eventually be Exported Transporters (SWEETs) and sucrose transporters or sucrose carriers (SUTs/SUCs). SWEET proteins act to export sucrose and hexose to the phloem apoplasm (6, 7), while SUT proteins mainly import sucrose from the apoplasm into the sieve tube symplasm (8–10). SUT proteins have 12 transmembrane domains that form a pore in the membrane to allow the passage of sucrose, and they utilize the energy stored in the proton gradient across the membrane generated by H^+^-adenosinetriphosphatases to drive sucrose transport (3, 11). In addition, cell wall invertases hydrolyze the unloaded sucrose from the phloem in the sink tissues into glucose and fructose, which are subsequently taken up by membrane-bound monosaccharide transporters of the recipient cells in developing tissues (5, 12). Disruption of the various sugar transporters often results in elevated sugar content in the source leaves and abnormal reproductive development (including abnormal anther development, impaired male fertility, and seed filling) because of carbohydrate deprivation (10, 13–17).

Given the important roles of sugar transport in plant growth and development, it is not surprising that the activities of various sugar transporters must be tightly regulated. Emerging evidence suggests that sugar transporters can be regulated at both the transcriptional and posttranscriptional levels (phosphorylation and dephosphorylation) (18). For example, the *Carbon Starved Anther* (*CSA*) gene encodes a putative R2R3 MYB-type transcription factor regulating the expression of *OsMST8* during male reproductive development (17), and the rate of carbon export from source leaves is controlled by ubiquitination and phosphorylation of SUC2 (19). There is also evidence indicating that biotic and abiotic factors (including light, water and salt stress, and pathogen attack) and phytohormones have an impact on the expression of sugar transporters (18, 20). For example, several studies have suggested that auxin signaling might be linked to sugar metabolism (4, 21). Down-regulation of the tomato *IAA9* gene and auxin response repressor *ARF4* leads to increased input of sugar to the fruit (22, 23). Despite the progress made in this area, however, the molecular mechanisms regulating carbohydrate partitioning between source and sink tissues remain elusive.

In a previous study, we reported that the rice *dao* mutant, which is defective in a gene encoding a 2-oxoglutarate-depenedent-Fe (II) dioxygenase, failed to convert indole-3-acetic acid (IAA) into 2-oxoindole-3-acetic acid (OxIAA), the important step toward the degradation of IAA (24, 25). The *dao* mutant displayed a pleiotropic reproductive phenotype, including closed spikelets, indehiscent anthers, and development of parthenocarpic seeds filled with a sucrose-rich liquid. In this study, we show that *DAO*-mediated auxin homeostasis plays an important role in regulating carbohydrate partitioning between the source and sink tissues to regulate reproductive organ development in rice.

## Results

### Elevated Auxin Levels Cause Closed Spikelets, Indehiscent Anthers, and Formation of Parthenocarpic Seeds.

**Fig. 1.**
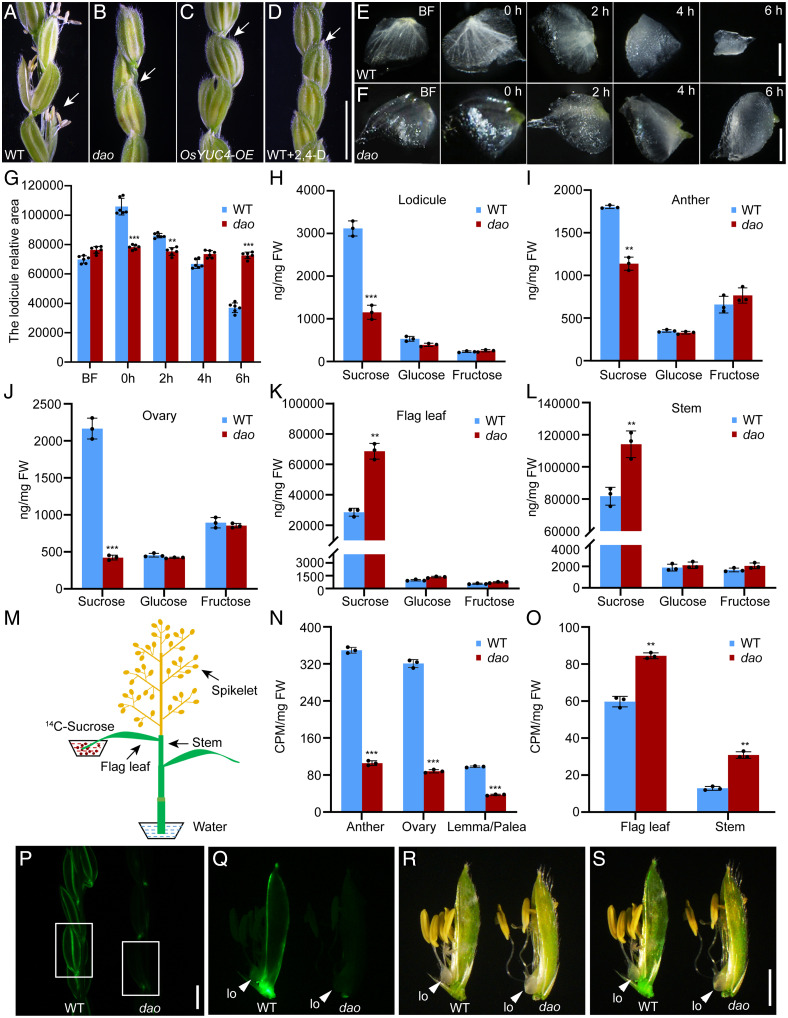
Phenotypic comparison of WT and *dao*, *OsYUC4-OE*, and WT plants treated with 2,4-D and measurement of sugar levels in the WT and *dao* mutants. (*A*–*D*) Comparison of panicles from WT (*A*), *dao* (*B*), *OsYUC4-OE* (*C*), and WT plants treated with exogenous 2,4-D (*D*) at the heading stage. Arrows indicate spikelet opening. (Scale bar, 5 cm.) (*E* and *F*) Morphological changes of lodicules of WT (*E*), and *dao* mutants (*F*) at different flowering times. (Scale bar, 10 µm.) BF indicates before flowering. (*G*) Changes in the sizes of the lodicule areas at different flowering time. Data shown are means ± SD (*n* = 6; **P < 0.01, ***P < 0.001, based on Student’s *t* test). (*H*–*L*) Sugar levels in lodicules (*H*), anthers (*I*), ovaries (*J*), flag leaves (*K*), and stems (*L*) of WT and *dao* mutants, respectively. Data shown are means ± SD (*n* = 3; **P < 0.01, ***P < 0.001, based on Student’s *t* test). (*M*) Schematic diagrams for ^14^C-sucrose feeding of flag leaves. (*N* and *O*) ^14^C-sucrose accumulation in the sink tissues (anthers, ovaries, and lemmas/paleas) (*N*) and flag leaves and stems (*O*) of WT and *dao* mutants, respectively. Data shown are means ± SD (*n* = 3; **P < 0.01, ***P < 0.001, based on Student’s *t* test). (*P*) Fluorescence microscopy examination of 5, 6-CFDA distribution (shown by the green fluorescence) in the panicles of the WT and *dao* mutants. The white boxes represent spikelets. (Scale bar, 10 cm.) (*Q*–*S*) 5, 6-CFDA fluorescence accumulation in the lodicules of WT and *dao* mutants. The lodicules are shown in the fluorescence (*Q*), in the bright-field (*R*), and in the merged image (*S*). Arrowheads indicate the lodicule (lo). (Scale bar, 10 cm.) CPM, counts per minute; FW, fresh weight.

We previously showed that the rice *dao* mutant displays parthenocarpic seeds due to elevated auxin levels (24). To test whether other reproductive defects (closed spikelets and indehiscent anthers) of the rice *dao* mutant are also caused by elevated auxin levels in these organs, we generated transgenic rice plants overexpressing *OsYUC4* (*SI Appendix*, Fig. S1 *A* and *B*), a member of the *OsYUC* (*YUCCA*) gene family that is highly expressed in rice anthers (26). The *YUC* family of flavin monooxygenases catalyzes the rate-limiting step in IAA biosynthesis (27). As expected, the *OsYUC4*-overexpressing plants exhibited a *dao*-like phenotype, including closed spikelets, indehiscent anthers, and parthenocarpic seeds. In addition, treatment of wild-type (WT) panicles with exogenous auxin (2,4-D) also led to similar phenotypes (Fig. 1 *A*–*D* and *SI Appendix*, Fig. S1 *C*–*J*). These results indicate that elevated auxin levels cause abnormal development of reproductive organs in rice.

### The Lodicules Fail to Enlarge at Anthesis in the *dao* Mutant.

The closed spikelets phenotype of the *dao* mutant, *OsYUC4* overexpressors, or WT plants treated with exogenous auxin led us to analyze lodicule development, which is a grass-specific floral organ with scale-like shapes that swell rapidly immediately before anthesis to promote spikelet opening, allowing subsequent pollination/fertilization (28–30). No significant difference was observed in the sizes of WT and *dao* mutant lodicules before flowering (Fig. 1 *E* and *F*). At anthesis, the size of lodicules in the WT was obviously larger than that in the *dao* mutant (Fig. 1*G*). After pollination, the lodicules in the WT became shrunken, leading to closed glumes. In contrast, the lodicules failed to expand during anthesis, and the glumes remained closed in the *dao* mutant (Fig. 1*G*).

### Abnormal Sugar Partition in the *dao* Mutant.

The failure of lodicules to expand at anthesis and the closed glume phenotype of the *dao* mutant prompted us to speculate that this phenotype might be caused by abnormal cell wall expansion of the lodicules, defects in water flux, and/or carbohydrate partitioning. Thus, we first analyzed the lodicule morphology of WT and *dao* mutants by scanning electron microscopy. No apparent differences were found in the cell wall of lodicules in WT and the *dao* mutant (*SI Appendix*, Fig. S2). As concentration of sucrose in the phloem is thought to be the dominant osmoticum that drives translocation of all other solutes, including water flux (3, 7), we measured the sugar content in the lodicules of WT and *dao* mutants. As expected, sucrose levels were significantly lower in the lodicules of *dao* compared to those in WT (Fig. 1*H*). In addition, we also measured the sugar contents in the sink tissues (anthers and ovaries), source tissues (flag leaves), and surrounding tissues (stems) of both WT and *dao* mutants. Strikingly, sucrose levels were significantly lower in the sink tissues of *dao* compared to those in WT. In contrast, higher levels of sucrose were found in the source tissues of the *dao* mutants compared to those in WT (Fig. 1 *I*–*L*). These observations indicate a potential defect in carbohydrate partitioning between the source and sink tissues in the *dao* mutant.

To directly test this possibility, we performed isotope-labeling experiments by feeding the flag leaves of WT and *dao* mutants with [^14^C] sucrose (Fig. 1*M*). Radioactivity testing showed that 24 h after treatment, the *dao* mutants had more labeled sucrose in the flag leaves and stems than the WT. In contrast, the WT had more [^14^C] label in the anthers and ovaries than the *dao* mutant plants (Fig. 1 *N* and *O*). Additionally, we fed the flag leaves of WT and *dao* mutants with the same concentration of 5, 6-carboxyfluorescein diacetate (5, 6-CFDA), a membrane-permeable, nonfluorescent dye (*SI Appendix*, Fig. S3*A*). Upon entering the cell, 5, 6-CFDA is converted into a fluorescent, membrane-impermeable fluorescent form (CF) tracer. In the symplasm, it can be translocated through the phloem (31). Half an hour after the treatment, strong fluorescence was detected in the sink tissues (panicles, spikelets, and lodicules) of WT but not of the *dao* mutant plants (Fig. 1 *P*–*S* and *SI Appendix*, Fig. S3 *B*–*D*). In addition, we submerged the excised stem of WT and *dao* mutant in water containing 5, 6-CFDA. After 30 min of treatment, the CF tracer was detected in the stem of both the WT and *dao* mutant (*SI Appendix*, Fig. S4 *A*–*F*). Moreover, we found that 5, 6-CFDA movement in the panicles and lodicules of WT plants treated with 2,4-D (auxin applied to the flag leaf) was inhibited by auxin treatment (*SI Appendix*, Fig. S4 *G*–*L*). Together, these results suggest that defects in sugar loading in the *dao* mutant are mainly caused by elevated auxin levels rather than defective phloem elements.

### Mutations in *Ossut1* Cause Defective Development of the Lodicule, Anther, and Ovary.

**Fig. 2.**
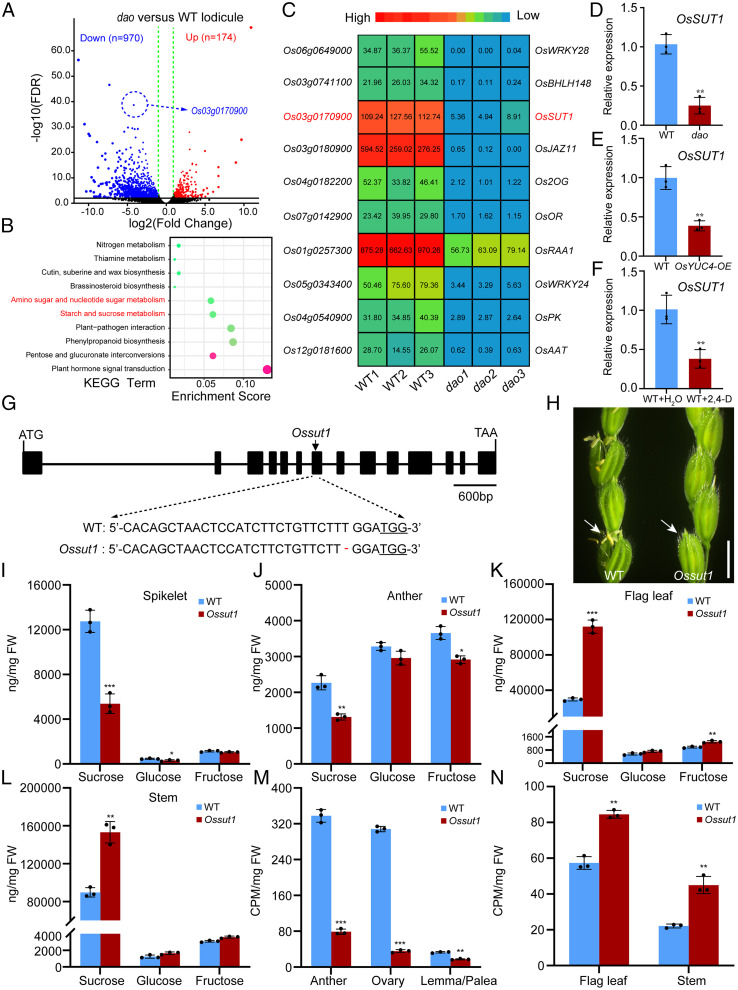
Identification of *OsSUT1* as a DEG in the lodicules of WT and *dao* mutant. (*A*) Volcano plot of DEGs extracted based on RNA-seq data from *dao* versus WT lodicules. The vertical dashed green lines represent the dividing line between down- and up-regulation of genes. Down, down-regulated genes; Up, up-regulated genes. (*B*) KEGG analysis of DEGs in the RNA-seq data. (*C*) Heat map of the 10 most significantly down-regulated DEGs in the lodicules of *dao* mutants. The color key (red to blue) represents gene expression FPKM as fold change. The gene-encoding proteins are shown on the right. (*D*–*F*) qRT-PCR analyses of the expression of *OsSUT1* in the WT and *dao* (*D*), *OsYUC4*-*OE* (*E*), and WT plants treated with 2,4-D and water (control) (*F*). Data shown are means ± SD (*n* = 3; *P < 0.05, **P < 0.01, based on Student’s *t* test). Ubiquitin (*LOC_Os03g13170*) was used as control. (*G*) CRISPR-Cas9–mediated knockout of *OsSUT1*. Filled boxes and lines represent exons and introns, respectively. Arrows indicate the site of the mutation in *Ossut1*. (*Bottom*) The lower panel shows alignment of WT and *Ossut1* sequences containing the CRISPR-Cas9 target sites. (*H*) Comparison of WT and *Ossut1* panicles. Arrows indicate spikelet opening. (Scale bar, 5 cm.) (*I*–*L*) Sugar levels in spikelet (*I*), anther (*J*), flag leaf (*K*), and stem (*L*) of WT and *Ossut1* mutants. Data are shown as means ± SD (*n* = 3; *P < 0.05, **P < 0.01, ***P < 0.001, based on Student’s *t* test). (*M* and *N*) ^14^C-sucrose accumulation in the sink tissues (anthers, ovaries, and lemmas/paleas) (*M*) and flag leaves and stems (*N*) of WT and *Ossut1* mutant. Data are shown as means ± SD (*n* = 3; **P < 0.01, ***P < 0.001, based on Student’s *t* test). FDR, false discovery rate; FW, fresh weight.

To investigate the molecular mechanisms of auxin-regulating source-sink carbohydrate partitioning in rice, we conducted RNA sequencing (RNA-seq) analysis of the lodicules in the WT and *dao* mutant (lodicules collected at 9:00 to 10:00 AM before flowering). A total of 1,144 differentially expressed genes (DEGs) were detected, among which 174 genes were up-regulated and 970 were down-regulated (Fig. 2*A*). Kyoto Encyclopedia of Genes and Genomes (KEGG) pathway (Fig. 2*B* and Dataset S1) and gene ontology analysis indicated enrichment of plant hormone signal transduction, carbon metabolism, starch, and sucrose metabolism in the DEGs (*SI Appendix*, Fig. S5 and Dataset S2). Notably, *Os03g0170900* (known as *OsSUT1*) was among the 10 most significantly down-regulated DEGs in the *dao* mutant (Fig. 2*C*). qRT-PCR verified this result (Fig. 2 *D*–*F* and *SI Appendix*, Fig. S6 *A*–*C*). These observations suggest that *DAO* and auxin play an important role in regulating *OsSUT1* expression.

To test whether the altered expression of *OsSUT1* might contribute to the observed phenotype of the *dao* mutant, we generated *OsSUT1* RNA interference knockdown transgenic plants (*SI Appendix*, Fig. S6*D*). These transgenic plants showed the spikelet closing and anther indehiscence phenotype (*SI Appendix*, Fig. S6 *E* and *F*). In addition, we obtained *Ossut1* knockout mutants using the CRISPR-Cas9 technique (Fig. 2*G*). As expected, the *Ossut1* knockout mutants exhibited a *dao*-like phenotype including closed spikelets, defective pollen germination, and formation of parthenocarpic seeds (Fig. 2*H* and *SI Appendix*, Fig. S6 *G*–*N*). Moreover, measurements of sugar content using gas chromatography–mass spectrometry (GC-MS) showed that the *Ossut1* mutant had significantly lower levels of sucrose in the sink tissues but significantly higher levels of sucrose in the source and surrounding tissues compared with the WT (Fig. 2 *I*–*L*). Feeding experiments with [^14^C] sucrose revealed results similar to those of GC-MS experiments (Fig. 2 *M* and *N*). These results demonstrate that *OsSUT1* plays a critical role in carbohydrate partitioning during rice reproductive development.

### Knocking Out *OsARF2* Phenocopies the *dao* Mutant.

**Fig. 3.**
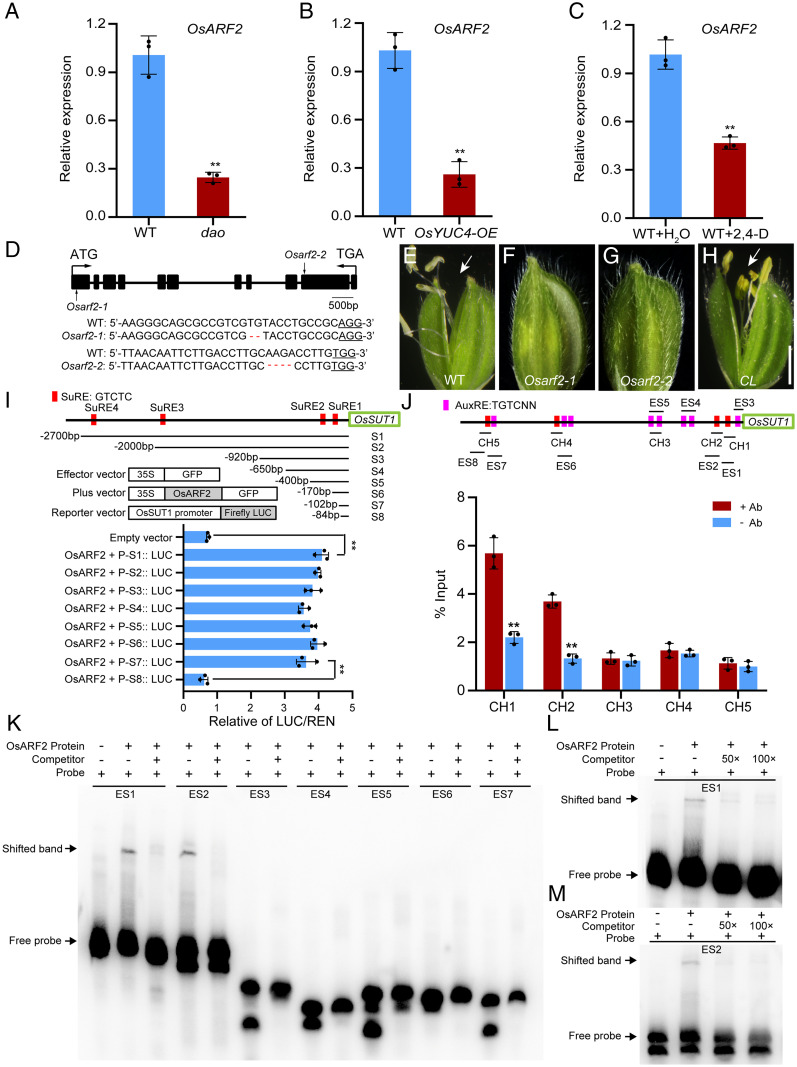
Functional characterization of OsARF2 and its direct binding to the *OsSUT1* promoter. (*A*–*C*) qRT-PCR analyses of *OsARF2* expression in WT and *dao* mutants (*A*), *OsYUC4*-*OE* (*B*), and WT plants treated with 2,4-D (*C*). Data shown are means ± SD (*n* = 3; **P < 0.01, based on Student’s *t* test). Ubiquitin (*LOC_Os03g13170*) was used as a control. (*D*) CRISPR-Cas9–mediated knockout of *OsARF2*. Filled boxes and lines represent exons and introns, respectively. Arrows indicate the site of mutations in *Osarf2-1* and *Osarf2-2*. (*Bottom*) The lower panel shows alignment of WT, *Osarf2-1*, and *Osarf2-2* sequences containing the CRISPR-Cas9 target sites. Red dashes represent the 2-bp and 4-bp deletions in the *Osarf2-1* and *Osarf2-2* mutants, respectively. (*E*–*H*) Comparison of spikelets in WT (*E*), *Osarf2-1* (*F*), *Osarf2-2* (*G*), and the complemented line (CL) of *Osarf2-1* (*H*). Arrows indicate spikelet opening. (Scale bar, 5 mm.) (*I*) Effects of OsARF2 coexpression on luciferase expression driven by various fragments of the *OsSUT1* promoter. *Top* indicates the positions of these fragments. *Bottom* shows the relative luciferase activity. Values are means ± SD (*n* = 3 biological replicates). Significance analysis was conducted using Student’s *t* tests (**P < 0.01). Red boxes indicate the positions of SuREs in the *OsSUT1* promoter. The green box represents the OsSUT1 coding sequence. (*J*) ChIP-qPCR assays of OsARF2 protein binding to the *OsSUT1* promoter. The fragments (CH1 to CH5) are indicated on the *OsARF2* promoter (*I*). Magenta boxes indicate the positions of the AuxRE motifs and red boxes indicate the positions of SuREs in the *OsSUT1* promoter. Values are means ± SD (*n* = 3 pooled tissues, five plants per pool; **P < 0.01, based on Student’s *t* test). (*K*–*M*) EMSA analysis of OsARF2 binding to the SuRE elements in the *OsSUT1* promoter. A series of probes were designed in the *OsSUT1* promoter (*K*). Two probes ES1 (*L*) and ES2 (*M*) containing the SuRE1 and SuRE2 elements, respectively, were bound by OsARF2.

Auxin is a major plant hormone that regulates many aspects of plant growth and development, including embryogenesis, seed development, root development, seedling growth, vascular patterning, and reproductive development (26, 32–34). The canonical auxin-signaling pathway is centered on activation or repression of gene expression by a family of auxin response factors (ARFs) (35, 36). The rice genome encodes 25 OsARF proteins (37). To identify the *OsARFs* that may contribute to the *dao* phenotypes, we analyzed the RNA-seq data of the 25 *OsARFs* in the lodicules of WT and the *dao* mutant (*SI Appendix*, Fig. S7*A*). We found that expression of *OsARF2* was significantly lower in the *dao* mutants (Fig. 3*A* and *SI Appendix*, Fig. S7*B*), suggesting that expression of *OsARF2* might be negatively regulated by the elevated level of auxin in the *dao* mutant. Consistent with this notion, *OsARF2* expression was significantly decreased in the *OsYUC4* overexpression lines and in WT panicles treated with exogenous auxin (Fig. 3 *B* and *C*, and *SI Appendix*, Fig. S7 *C* and *D*). qRT-PCR experiments demonstrated that *OsARF2* was mainly expressed in stems, young anthers, and developing ovaries (*SI Appendix*, Fig. S7*E*).

To test whether *OsARF2* plays a role in regulating reproductive development, we performed targeted mutagenesis of *OsARF2* in the ZH11 cultivar background using CRISPR-Cas9 technology. We obtained two independent, *Cas9*-free, homozygous *Osarf2* mutants, *Osarf2-1* and *Osarf2-2* (Fig. 3*D*). No significant differences were observed between the *Osarf2-1* mutant and WT plants during vegetative growth. As in the *dao* mutant, some glumes of the *Osarf2-1* mutants failed to open normally at the anthesis stage (Fig. 3 *E*–*G*). In addition, some anthers remained completely indehiscent and did not release mature pollen grains, and some seeds were parthenocarpic (*SI Appendix*, Fig. S7 *F*–*H*). To further confirm that these phenotypes were caused by mutations in the *OsARF2* gene, we performed a functional complementation experiment. We introduced a 6.8-kb WT DNA fragment containing the *OsARF2* promoter and its coding region into the *Osarf2* mutant. The transgene fully rescued the *Osarf2* mutant phenotypes, including spikelet opening, anther dehiscence, and seed development (Fig. 3*H* and *SI Appendix*, Fig. S7*I*). Collectively, these results indicate that *OsARF2* plays an essential role in regulating spikelet opening, anther dehiscence, and seed development.

### OsARF2 Directly Regulates the Expression of *OsSUT1* via Binding to the Sugar-Responsive Elements (SuREs).

ARF proteins are known to regulate downstream gene expression by binding to the cis-element motifs termed auxin-responsive elements (AuxREs: TGTCNN) in the target gene promoters using a conserved DNA-binding domain at the N terminus (38–41). We identified eight AuxREs and four sugar-responsive elements (SuRE1–SuRE4: GTCTC) (42) in the *OsSUT1* promoter region (Fig. 3 *I* and *J*). Interestingly, the sequences of AuxREs and SuREs are highly similar. To test whether OsARF2 directly regulates *OsSUT1* expression, we prepared various deletion fragments of the *OsSUT1* promoter (from −2,700 bp to −84 bp), fused them to the luciferase reporter gene coding sequence, and performed transient expression experiments in rice protoplasts. LUC activity was significantly up-regulated to a comparable level when *OsARF2* was coexpressed with the P-S1::LUC to P-S7::LUC reporters, but not with the P-S8::LUC reporter (Fig. 3*I*), suggesting that OsARF2 may regulate *OsSUT1* expression by direct binding to the SuRE1 or SuRE2 element, both of which are present in the S1-S7 fragments but missing in the S8 fragment. Chromatin immunoprecipitation (ChIP)–qPCR assays showed that, indeed, OsARF2 could directly bind to the CH1 and CH2 fragments that contain the SuRE1 or SuRE2 element, respectively, but not to other fragments (Fig. 3*J*). Moreover, electrophoretic mobility shift assay (EMSA) also showed that OsARF2 could directly bind to the SuRE1 and SuRE2 fragments (Fig. 3*K*), and the binding activity was gradually reduced by increased concentrations of unlabeled probes (Fig. 3 *L* and *M*). Further, mutation or deletion of the SuRE1 and SuRE2 elements reduced or abolished the binding activity of OsARF2 (*SI Appendix*, Fig. S8 *A*–*C*). These results verify that OsARF2 directly regulates *OsSUT1* expression through direct binding to the SuRE1 and SuRE2 elements in the *OsSUT1* promoter.

The observation that *OsARF2* directly binds to the SuRE1 and SuRE2 elements but not the AuxRE elements in the *OsSUT1* promoter (Fig. 3*K*) suggested to us that SuREs might represent a novel type of ARF-binding sites or that the DNA-binding activity of ARF proteins might be promiscuous. In order to differentiate these possibilities, we tested binding of OsARF2 to the promoters of other sucrose transporters, including *OsSUT2*, *OsSUT3*, *OsSUT4*, and *OsSUT5*. Sequence analysis revealed that there are two SuRE elements in the *OsSUT2* promoter, one in the *OsSUT3* promoter, one in the *OsSUT4* promoter, and two in the *OsSUT5* promoter (*SI Appendix*, Fig. S9*A*). Yeast one-hybrid assay showed that OsARF2 could also bind to the promoters of *OsSUT4* and *OsSUT5*, but not to other*s* (*SI Appendix*, Fig. S9*A*). Consistent with these results, qRT-PCR experiments showed that the expression of *OsSUT4* and *OsSUT5* was also down-regulated in the *Osarf2* mutant (*SI Appendix*, Fig. S9*B*). These observations suggest that in addition to *OsSUT1*, *OsARF2* might also regulate the expression of *OsSUT4* and *OsSUT5* through direct binding to the SuRE elements in their promoters.

**Fig. 4.**
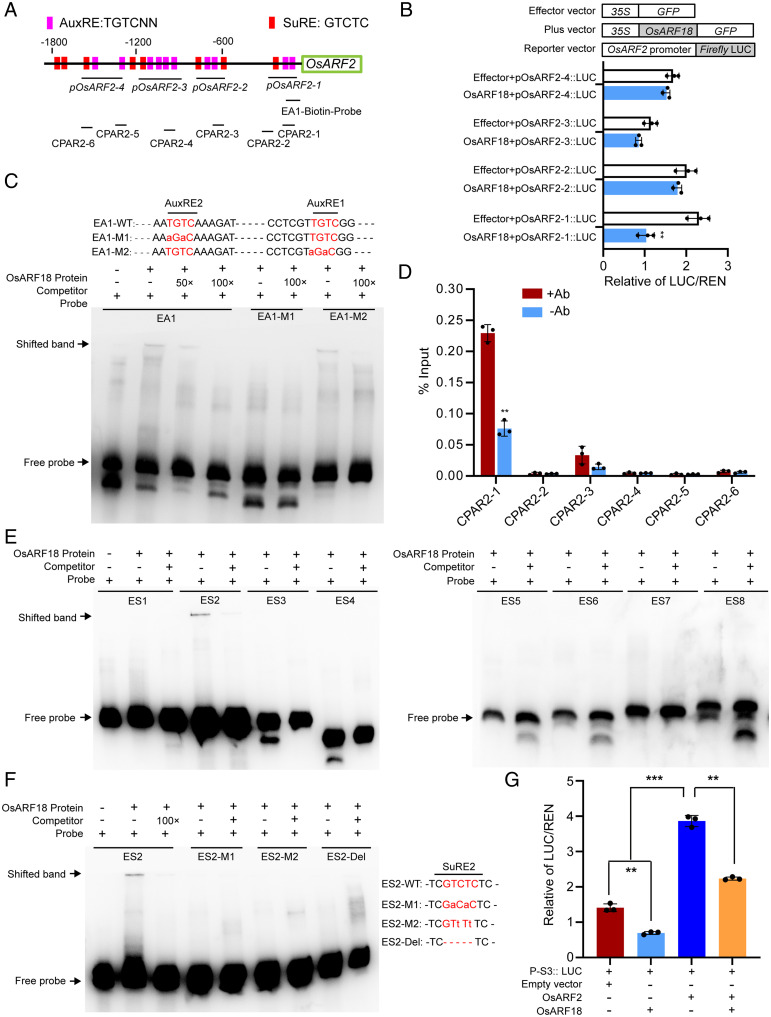
OsARF18 negatively regulates *OsARF2* and *OsSUT1* expression. (*A*) Diagram of the *OsARF2* promoter region. Magenta boxes represent the positions of the AuxRE elements, and the red boxes represent the positions of the SuRE motifs in the *OsARF2* promoter. (*B*) Effects of OsARF18 coexpression on luciferase expression driven by various *OsARF2* promoter fragments. Locations of these various fragments are indicated in the *OsARF2* promoter region in *A*. Values are means ± SD (*n* = 3 biological replicates). Significance analysis was conducted with Student’s *t* tests (**P < 0.01). (*C*) EMSA shows that the GST-OsARF18 recombinant protein directly binds to the EA1 fragment of the *OsARF2* promoter. The AuxREs are labeled in red. (*D*) ChIP-qPCR assays of OsARF18 protein binding to the *OsARF2* promoter. The positions of various fragments (CPAR2-1 to CPAR2-6) on the *OsARF2* promoter were shown in *A*. Values are means ± SD (*n* = 3 pooled tissues, five plants per pool; **P < 0.01, based on Student’s *t* test). (*E*) EMSA shows that OsARF18 binds to the ES2 fragment of the *OsSUT1* promoter. (*F*) EMSA shows that OsARF18 binds to the WT ES2 fragments but not the fragments with mutated SuRE2. The SuRE2 elements are labeled in red. (*G*) Effects of *OsARF18* and *OsARF2* coexpression on luciferase expression driven by *OsSUT1* promoter fragments P-S3 containing the SuRE2 element. Values are means ± SD (*n* = 3 biological replicates). Significance analysis was conducted with Student’s *t* tests (**P < 0.01, ***P < 0.001, based on Student’s *t* test).

To further delineate the nucleotides in the SuRE motifs for specific binding of OsARF2, we conducted mutagenesis of each of the five nucleotides in the SuRE1 motif of the *OsSUT1* promoter. Results showed that mutations of the first G, fourth T, or fifth C in the SuRE1 motif dramatically impaired the binding activity of OsARF2, suggesting that these three nucleotides are essential for specific binding of OsARF2 (*SI Appendix*, Fig. S8 *D* and *E*). Interestingly, we also identified 8 SuRE and 10 AuxRE elements in the *OsARF2* promoter (Fig. 4*A*), raising the possibility that OsARF2 might also regulate its own expression. EMSA experiments showed that OsARF2 cannot bind to the AuxRE elements but directly binds to the SuRE2 and SuRE7 elements in its own promoter, and the binding activity was gradually reduced by increased concentrations of unlabeled probes (*SI Appendix*, Fig. S10 *A* and *B*). Furthermore, luciferase reporter gene assay showed that OsARF2 could positively regulate its own expression (*SI Appendix*, Fig. S10*C*). These results showed that OsARF2 activates the expression of *OsSUT1* and itself by direct binding to the SuRE motifs in their promoters.

### OsARF18 Negatively Regulates *OsARF2* and *OsSUT1* Expression.

*OsARF18* encodes a putative transcriptional repressor (37). A previous study reported that expression of *OsARF2* was decreased in *OsARF18* overexpression lines and that the *OsARF18* overexpressors were defective in reproductive development, including abnormal flower and seed development (43). qRT-PCR analysis showed that *OsARF18* expression was significantly up-regulated in the *dao* mutants, *OsYUC4* overexpressors, and WT panicles treated with exogenous auxin (*SI Appendix*, Fig. S11 *A*–*C*), suggesting that the expression of *OsARF18* was induced by high levels of auxin. qRT-PCR and the histochemical staining of *OsARF18::GUS* (β-glucuronidase) reporter lines showed that the expression of *OsARF18* was mainly expressed in stems, young anthers, and fertilized ovaries (*SI Appendix*, Fig. S11 *D*–*I*). To further test the role of *OsARF18* in regulating reproductive development, we generated *OsARF18* overexpression lines in the WT background. We did not observe significant differences between the WT and *OsARF18*-*OE* plants during the vegetative growth period, except that the *OsARF18*-*OE* plants were shorter and had fewer tillers (*SI Appendix*, Fig. S12*A*). At the heading stage, the *OsARF18*-*OE* plants developed closed glumes and indehiscent anthers and produced parthenocarpic seeds (*SI Appendix*, Fig. S12 *B*–*H*).

To explore the relationship between *OsARF18* with *OsARF2* and *OsSUT1* in regulating rice reproductive development, we compared sugar contents in the source and sink tissues of WT, *OsARF18-OE*, and *Osarf2* mutant plants. Compared to WT, the sucrose levels in the sink tissues of *OsARF18-OE* and *Osarf2* were all significantly decreased; however, higher levels of sucrose accumulated in the source tissues in the *OsARF18-OE* and *Osarf2* mutant plants (*SI Appendix*, Fig. S13 *A*–*D*). Moreover, qRT-PCR analysis showed that expression of both *OsARF2* and *OsSUT1* was significantly decreased in the *OsARF18*-*OE* and *Osarf2* mutant plants compared to that in the WT control (*SI Appendix*, Figs. S12*H* and S13 *E* and *F*). These observations suggest that *OsARF18* might regulate rice reproductive development by repressing expression of *OsARF2* and *OsSUT1*.

To test whether *OsARF18* directly regulates *OsARF2* expression, we performed transient expression experiments of a series of *LUC* reporter genes driven by different promoter fragments of *OsARF2* (ProARF2-1 to ProARF2-4) in rice protoplasts. The LUC activity of ProARF2-1::LUC was significantly decreased, while the other three reporters (ProARF2-2::LUC, ProARF2-3::LUC, and ProARF2-4::LUC) had no significant change when coexpressed with *OsARF18* (Fig. 4*B*). In addition, EMSA showed that OsARF18 directly bound to the EA1 fragment containing the AuxRE1 and AuxRE2 elements and this binding was gradually diminished by increased concentrations of unlabeled competitive probes (Fig. 4*C*). In addition, mutations of the AuxRE2 element abolished the binding activity of OsARF18, while mutations of AuxRE1 had no effect (Fig. 4*C*), suggesting that AuxRE2 is responsible for specific OsARF18 binding. Consistent with this, ChIP-PCR assay validated that OsARF18 could directly bind to various fragments of the *OsARF2* promoter containing the AuxRE elements but not to fragments lacking the AuxRE motifs (Fig. 4*D*). Taken together, these results indicate that OsARF18 directly binds to the AuxRE2 element of the *OsARF2* promoter and represses its expression.

Similarly, EMSA showed that OsARF18 could directly bind to the ES2 fragment containing the SuRE2 element in the *OsSUT1* promoter and that the binding activity was gradually reduced by increased concentrations of unlabeled probes (Fig. 4*E*). Mutation or deletion of the SuRE2 element abolished the binding activity of OsARF18 (Fig. 4*F*), indicating that the binding was specific. As OsARF2 could also bind to the SuRE2 element to up-regulate the expression of *OsSUT1* (Fig. 3*M*), we performed transient expression experiments in *Nicotiana benthamiana* to test the combined effect of OsARF2 and OsARF18 on the expression of *OsSUT1*. Coexpression of individual *OsARF18* or *OsARF2* with the P-S3::LUC reporter containing the SuRE2 element significantly decreased or increased the LUC activity of the reporter gene, respectively. When both *OsARF18* and *OsARF2* were coexpressed with the P-S3::LUC reporter, LUC activity was also significantly decreased (Fig. 4*G*). Taken together, these results suggest that OsARF18 could compete with OsARF2 for binding to the SuRE2 element in the *OsSUT1* promoter, thus repressing *OsSUT1* expression.

**Fig. 5.**
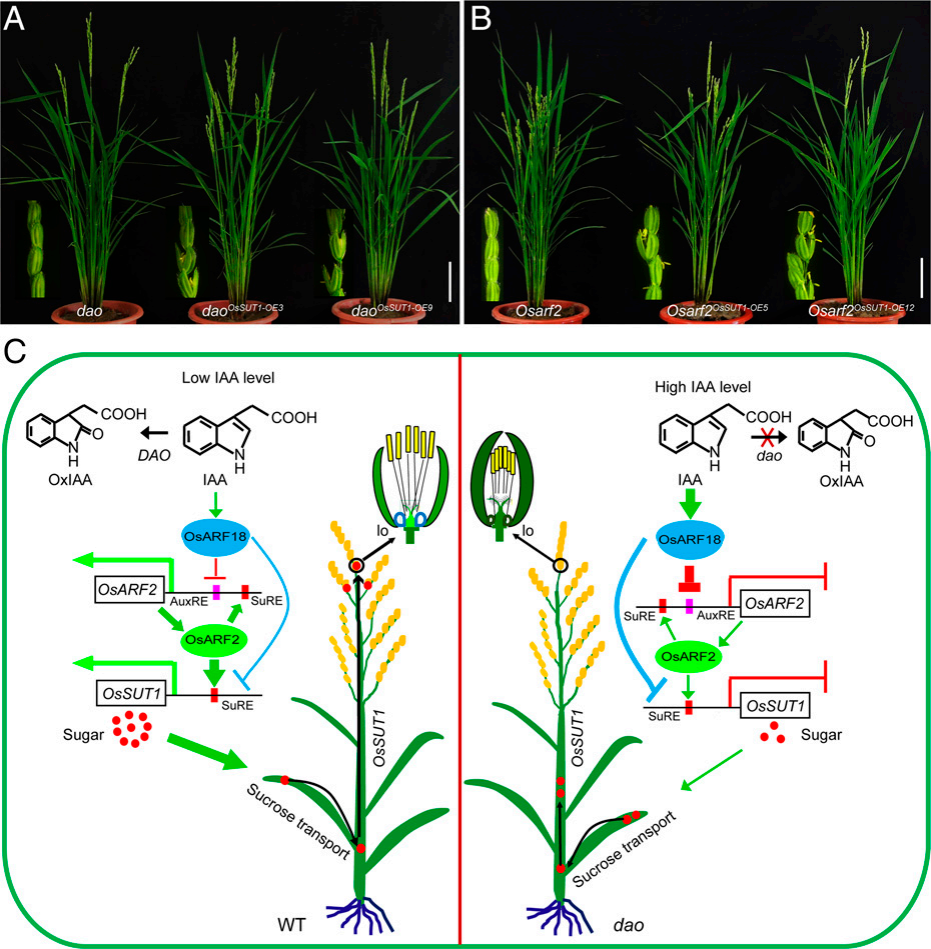
Overexpression of *OsSUT1* in the *dao* and *Osarf2* mutant backgrounds partially rescues the spikelet opening defect. (*A* and *B*) Overexpression of *OsSUT1* in the *dao* and *Osarf2* mutant backgrounds. (*A*) The panicles of *dao* mutant and *dao* transgenic plants overexpressing *OsSUT1* at the heading stage. (*B*) The panicles of *Osarf2* mutant and *Osarf2* transgenic plants overexpressing *OsSUT1* at the heading stage. (Scale bars, 5 cm.) (*C*) A proposed model of auxin signaling regulating carbohydrate partitioning in rice reproductive development. In late rice reproductive developmental stages, low IAA levels are maintained by DAO-mediated IAA degradation. Low levels of IAA weakly activate OsARF18. Consequently, the transcriptional activator OsARF2 up-regulates its own expression by directly binding to the SuRE motifs in its own promoter; at the same time, it directly activates the expression of *OsSUT1* by binding to the SuRE motifs in the *OsSUT1* promoter. Sucrose is then transported via the phloem from flag leaves to the reproductive tissues, where it provides energy for flowering. In the *dao* mutant plant, IAA could not be converted into OxIAA, and the higher IAA levels strongly activated OsARF18, which repressed the expression of *OsARF2* and *OsSUT1* by directly binding to the AuxRE and SuRE motifs in their promoter, respectively. Subsequently, carbohydrate partitioning was also repressed, resulting in failure of spikelet opening. Magenta boxes indicate the positions of the AuxRE motifs and red boxes indicate the positions of SuREs in OsARF2 and OsSUT1 promoters. lo, lodicule.

Finally, to substantiate the notion that reduced *OsSUT1* expression contributes to reproductive defects in the *dao* and *Osarf2* mutants, we overexpressed *OsSUT1* in the *dao* and *Osarf2* mutant backgrounds, respectively. Compared with these mutants, the spikelet opening and seed setting rates of the overexpressed transgenic plants were both significantly increased (Fig. 5 *A* and *B*, and *SI Appendix*, Fig. S14). Together, these results suggest that auxin plays an important role in regulating carbohydrate partitioning during rice reproductive development by regulating *OsSUT1* expression.

## Discussion

Understanding the molecular mechanisms regulating source-sink carbohydrate partitioning is a fundamental question in plant biology, which also bears important implications for increasing crop yields (3–5). Although it has long been recognized that long-range transport of auxin and sugar from the source to sink tissues is important for growth and developmental processes, such as lateral root development, hypocotyl elongation, and shoot branching, little is known about the interplay between auxin and sugar (5, 34, 44). In this study, we uncovered an auxin-signaling cascade regulating carbohydrate partitioning during rice reproductive development. Several lines of evidence were collected. First, we showed that the *dao* mutant, *OsYUC4* overexpressors, or WT plants treated with exogenous auxin are all defective in sucrose transport from the source tissues to the sink tissues, resulting in overaccumulation of sucrose in the source tissues (flag leaves) and stems and concomitant reduction of sucrose content in the sink tissues (lodicules, anthers, and ovaries) (Fig. 1 *H*–*L*). Second, we showed that the expression levels of *OsSUT1* are significantly reduced in the *dao* mutant, *OsYUC4* overexpressors, and WT plants treated with exogenous auxin (Fig. 2 *D*–*F*). Third, we showed that knockout mutants of *Ossut1, Osarf2*, and *OsARF18-OE* plants display a *dao*-like mutant phenotype and that they are defective in sucrose transport between the source and sink tissues during rice reproductive development (Fig. 2 *G* and *H* and *SI Appendix*, Fig. S6 *G*–*L* and S12). Fourth, we showed that OsARF2 directly activates the expression of *OsSUT1* and itself via binding to distinct SuRE elements in the *OsSUT1* and its own promoter (Fig. 3 *I*–*M* and *SI Appendix*, Fig. S10). Fifth, we showed that OsARF18 directly represses the expression of *OsARF2* and *OsSUT1* by binding a highly similar AuxRE2 element (TGTC) and SuRE2 element (GTCTC) in their promoters, respectively (Fig. 4 and *SI Appendix*, Figs. S12*H* and S13*E*). Sixth, we showed that overexpression of *OsSUT1* in the *dao* and *Osarf2* mutant backgrounds significantly improves spikelet opening and seed-setting defects of these mutants (Fig. 5 *A* and *B*). Based on these findings, we propose an OsARF18-OsARF2-OsSUT1–mediated auxin signaling cascade regulating carbohydrate partitioning between the source and sink tissues in rice, which is essential for proper development of rice reproductive organs, including lodicules, anthers, and seeds (Fig. 5*C*). Thus, our findings represent a major step forward in elucidating the regulatory relationship between auxin and sugar in regulating plant growth and development.

It should be noted that previous studies have reported that flowers of the *Arabidopsis arf6 arf8* double-null mutants also fail to open due to lack of rapid swelling and enlargement of the petals at anthesis. These mutants also have undehisced anthers that do not release pollens (45). These phenotypes are remarkably similar to the closed glumes and undehisced anthers phenotype exhibited in the *dao* mutant, *OsYUC4* overexpressors, and *Osarf2* mutants. It has been proposed that the lodicules in grass plants are morphologically equivalent to petals in dicot plants and play an important role in flower opening (28–30). Thus, we speculate that a similar auxin signaling cascade may operate in both dicot and monocot plants to regulate flower opening and anther dehiscence.

Our results may also have important implications for breeding crops with increased yields. Several recent studies have demonstrated that manipulation of source activity or sink strength could significantly enhance crop productivity (4, 21). For example, overexpression of the transcription factor *Higher Yield Rice* (*HYR*) associated with photosynthetic carbon metabolism promotes efficiencies in CO_2_ assimilation and photosynthesis in leaves and improves grain yield in rice (46). Engineering of synthetic glycolate metabolism pathways or new chloroplastic photorespiratory bypass (GOC bypass: Glycolate oxidase, Oxalate oxidase and Catalase) increases photosynthetic efficiency and yield potentials in the field (47, 48). However, the engineered GOC plants still suffer a low seed-setting rate. Thus, a major bottleneck to realizing grain yield increase is successful partitioning of photosynthates from the source tissues into the sink tissues. Our findings that auxin regulates carbohydrate partitioning during rice reproductive development may offer a viable approach to overcome this problem by better coordinating source and sink activities in crops.

## Methods

### Plant Materials and Growth Conditions.

The background of various transgenic plants (*OsYUC4-OE* and *OsARF18-OE*) and CRISPR-Cas9 knockout plants (*Osarf2-1*, *Osarf2-2*, and *Ossut1*) used in this study is *Oryza sativa* subsp. *japonica* cv. Zhonghua 11 (ZH11). The background of *dao* mutant is *O. sativa* subsp. *japonica* cv. Nipponbare. All plants were grown in the Tu Qiao Experiment Station of Nanjing Agricultural University, Nanjing, China. All materials were planted at a spacing of 16.5 cm × 16.5 cm. A wide-row spacing of 23.5 cm was set between the plots.

### In Vitro Pollen Germination.

Pollen grains of WT and mutants were germinated on 1% agar medium containing 15% (wt/vol) sucrose and 20 mg/L K_2_B_4_O_7_. Spikelets were sampled just after anthesis and gently shaken above germination medium on a slide to collect pollen grains. The slide was placed in an incubator for 30 min at 28 °C. Then, the number of germinated pollen grains was counted under a microscope.

### GC-MS Soluble Sugar Assays.

Soluble sugar levels were analyzed essentially as previously described (17, 49). Materials including fresh anthers, spikelets, lemma/paleas, flag leaves, or stems were immediately frozen in liquid nitrogen and ground into powder. Six hundred microliters of methanol was added to the powder and vortexed briefly, then 50 μL ribitol (0.2 ng/mL, as an internal standard; Sigma-Aldrich) was added. The extracts were centrifuged at 10,000×*g* for 2 min, and the supernatant was immediately transferred to a new centrifuge tube and dried under vacuum for sugar assays. Acetylated derivatives of the sugars were made by mixing dry weight powder of plant sample in Me_2_SO with 150 μL acetic anhydride and 30 μL 1-methylimidazole and stirring for 10 min in glass tubes. Six hundred microliters of double-distilled H_2_O was added to the tubes to remove the excess acetic anhydride. Then, 100 μL CH_2_Cl_2_ was added to extract the acetylated derivatives. The tubes were centrifuged for 1 min to partition the organic phases. Finally, 1 μL of the lower methylene chloride layer was injected for GC-MS analysis.

A GC-MS system (Trace-GC Ultra) connected to a Trace-DSQ mass selective detector with electrospray ionization set at full scan and selected ion monitoring (1.0 mass unit) (Thermo-Finnigan) was used to analyze the acetyl-derivatized sugars. The column used was a DB-17MS fused silica capillary column (30 m × 0.25 mm, 0.25-μm film thickness; Agilent). The GC oven temperature was programmed as follows: initiation at 100 °C, then gradually ramping to 190 °C (12 °C/min) and holding for 6 min, subsequent ramping to 250 °C (30 °C/min) and holding for 6 min, and finally ramping to 280 °C (40 °C/min) and holding for 10 min. The injector and detector port temperatures were set at 250 °C and 260 °C, respectively. Helium was used as the carrier gas at a constant flow rate of 1.0 mL/min. An Xcalibur 2.0 workstation was used for data acquisition and quantification.

### 5, 6-CFDA Feeding Experiments.

To examine sucrose uptake in the panicles of WT and mutant plants, feeding experiments using 5, 6-CFDA, a low-molecular-weight dye, were carried out. The WT and mutant panicles were placed in an aqueous solution containing 5, 6-CFDA (100 μg/μL). After 30 min in the dark, the WT and mutant panicles were examined under fluorescence microscopy.

### Radiolabeling Experiment.

Sucrose radiolabeling was performed essentially as described (17, 50). The WT and mutant flag leaves were placed in water containing 1 μCi [^14^C] sucrose (21.8 GBq mmol-1 in ethanol:water [9:1, vol/vol]; MP Biochemicals). The stems were kept in this solution for 24 h at room temperature, using the stem submerged in water without ^14^C-labeled sucrose as the control. Then, the anthers, ovaries, lemmas, paleas, flag leaves, and stems were collected for analysis. These materials were cut into pieces and incubated in 3 mL scintillation fluid. Radioactivity (counts per minute) was measured by liquid scintillation counting using a Beckman LS650. At least three biological replicates were measured.

### RNA Isolation and qRT-PCR Analysis.

Total RNAs were extracted from lodicules, anthers, ovaries, and flag leaves using TRIzol reagent (Invitrogen). Complementary DNAs were generated by reverse-transcription according to the manufacturer’s instructions (Invitrogen). qRT-PCR was carried out using gene-specific primers and SYBR Premix EX-Taq on a real-time PCR 7500 system (Applied Biosystems). Data were collected using the ABI Prism 7500 sequence detection system following the manufacturer’s instructions. The rice Ubiquitin gene (*LOC_Os03g13170*) was used as the control. At least three biological replicates and three technical repeats were conducted. All primers for qRT-PCR can be found in *SI Appendix*, Table S1.

### RNA-seq Analysis.

Before flowering, total RNAs were extracted from the lodicules of WT and *dao* mutant using TRIzol and were purified using QIAGEN RNeasy Mini kits. RNA-seq libraries were prepared from WT and *dao* mutant with three replicates. The libraries were sequenced using Illumina HiSeq X-Ten platform (Biomarker Biotechnology Co.), and generated reads were cleaned and then mapped to the reference genome (Nipponbare). After normalization, gene expression levels were estimated by fragments per kilobase of transcript per million fragments mapped (FPKM). The multiple-testing *P* value <0.01 and fold change >2 were used to determine whether the gene was significantly differentially expressed or not.

### Complementation of the *Osarf2-1* Mutant.

For functional complementation, an ∼6.8-kb fragment of genomic DNA containing the *OsARF2* promoter region and the entire coding region was subcloned into the binary vector pCAMBIA1305 carrying a hygromycin resistance marker to generate the p1305-OsARF2 construct. We induced homogenous *Osarf2-1* calli, which were then transformed by cocultivation with *Agrobacterium tumefaciens* strain EHA105 carrying the p1305-OsARF2 plasmid and the control plasmid p1305.

### *OsARF18::GUS* Reporter Gene Construct and Histochemical Staining.

A 2.5-kb genomic fragment containing the promoter upstream of the ATG start codon of *OsARF18* was amplified by PCR (primer sequences listed in *SI Appendix*, Table S1) using Nipponbare genomic DNA as the template and was cloned into the binary vector pCAMBIA1305 to drive expression of the *GUS* reporter gene. Transgenic plants were generated as described above. Images were captured using Leica Application Suite 3.3 and merged and enhanced using Photoshop CS (Adobe).

### Dual-Luciferase Reporter Assay.

To construct the effector plasmid, the full-length coding sequences of *OsARF18* and *OsARF2* were inserted into the vector pCAMBIA1305.1–green fluorescent protein (GFP). For the reporter construct, a 1,800-bp upstream fragment of *OsARF2* and a 920-bp upstream fragment of *OsSUT1* were inserted into pGreenII0800-LUC to drive the *firefly luciferase* (*LUC*) gene to generate the *ARF2* pro:LUC and *SUT1*pro:LUC constructs, respectively. These vectors were individually transformed into the *A. tumefaciens* strain EHA105. For transient coexpression, the effector and reporter constructs were coinfiltrated into the leaves of 4-week-old *N. benthamiana* plants for 2 d. Firefly LUC and REN activities were surveyed with a Dual–Luciferase Reporter Assay Kit (Promega), and the LUC activity, normalized to REN activity, was determined. The *Renilla luciferase* (*REN*) gene driven by 35S promoter was used as a normalization control. Transient transactivation with the reporter and the empty vector pCAMBIA1305.1-GFP was used as a negative control. Three biological replicates were performed for each assay.

### Purification of Recombinant Protein and EMSAs.

EMSA was performed using a light-shift chemiluminescent EMSA Kit (Pierce, 20148) following the manufacturer’s protocol. The *N*-termini of OsARF18 and OsARF2 recombinant protein were fused in frame with glutathione *S*-transferase (GST) and expressed in the BL21 *Escherichia coli* strain. The fused proteins were induced with 0.5 mM isopropyl B-D-1-thiogalactopyranoside and incubated at 16 °C for 28 h. The recombinant proteins were purified by GST-agarose affinity. The fragments of the *OsARF2* and *OsSUT1* promoter were amplified by PCR and labeled with and without 5′-biotin, respectively. The unlabeled fragments were used as competitors, and CKS-GST protein was used as a negative control.

### ChIP Assay.

The ChIP-qPCR assay was performed as described previously (51). Transgenic lines overexpressing *OsARF18-Flag* and *OsARF2-Flag* were used in this assay. ChIP was performed on flag leaves at the heading stage. The harvested samples were ground in liquid nitrogen. The powder was resuspended in buffer (50 mM *N*-2-hydroxyethylpiperazine-*N*′-2-ethanesulfonic acid [Hepes], pH 7.5, 150 mM KCl, 5 mM MgCl_2_, and 0.1% Triton X-100) supplemented with 1% formaldehyde and incubated for 10 min at 4 °C. The powder was then incubated with 0.15 M glycine for 5 min at 4 °C to quench the formaldehyde. The chromatin was subsequently isolated and sonicated to produce DNA fragments of around 300 bp. A flag tag–specific monoclonal antibody (Sigma-Aldrich, F1804; 1:300 dilution) was used for ChIP analysis. WT plants treated in the same way served as controls. The ChIP DNA products were analyzed by PCR using primers that were synthesized to amplify about 150-bp DNA fragments in the promoter region of *OsARF2* or *OsSUT1*. All PCR experiments were performed under the following conditions: 95 °C for 5 min, 40 cycles of 95 °C for 10 s, 60 °C for 25 s, and 72 °C for 20 s. All primer sequences used for ChIP assays are listed in *SI Appendix*, Table S1. The experiment was repeated three times.

## Supplementary Material

Supplementary File

Supplementary File

Supplementary File

## Data Availability

All study data are included in the article and/or supporting information.

## References

[1] K. Koch, Sucrose metabolism: Regulatory mechanisms and pivotal roles in sugar sensing and plant development. Curr. Opin. Plant Biol. 7, 235–246 (2004). 15134743 10.1016/j.pbi.2004.03.014

[2] J. Lastdrager, J. Hanson, S. Smeekens, Sugar signals and the control of plant growth and development. J. Exp. Bot. 65, 799–807 (2014). 24453229 10.1093/jxb/ert474

[3] D. M. Braun, L. Wang, Y. L. Ruan, Understanding and manipulating sucrose phloem loading, unloading, metabolism, and signalling to enhance crop yield and food security. J. Exp. Bot. 65, 1713–1735 (2014). 24347463 10.1093/jxb/ert416

[4] S. M. Yu, S. F. Lo, T. D. Ho, Source-sink communication: Regulated by hormone, nutrient, and stress cross-signaling. Trends Plant Sci. 20, 844–857 (2015). 26603980 10.1016/j.tplants.2015.10.009

[5] Y. L. Ruan, Sucrose metabolism: Gateway to diverse carbon use and sugar signaling. Annu. Rev. Plant Biol. 65, 33–67 (2014). 24579990 10.1146/annurev-arplant-050213-040251

[6] L. Q. Chen , Sucrose efflux mediated by SWEET proteins as a key step for phloem transport. Science 335, 207–211 (2012). 22157085 10.1126/science.1213351

[7] J. S. Eom , SWEETs, transporters for intracellular and intercellular sugar translocation. Curr. Opin. Plant Biol. 25, 53–62 (2015). 25988582 10.1016/j.pbi.2015.04.005

[8] L. Q. Chen , Sugar transporters for intercellular exchange and nutrition of pathogens. Nature 468, 527–532 (2010). 21107422 10.1038/nature09606 PMC3000469

[9] T. L. Slewinski, R. Meeley, D. M. Braun, Sucrose transporter1 functions in phloem loading in maize leaves. J. Exp. Bot. 60, 881–892 (2009). 19181865 10.1093/jxb/ern335 PMC2652052

[10] C. Kühn, C. P. L. Grof, Sucrose transporters of higher plants. Curr. Opin. Plant Biol. 13, 288–298 (2010). 20303321 10.1016/j.pbi.2010.02.001

[11] J. Doidy , Sugar transporters in plants and in their interactions with fungi. Trends Plant Sci. 17, 413–422 (2012). 22513109 10.1016/j.tplants.2012.03.009

[12] L. Wang, Y. L. Ruan, Critical roles of vacuolar invertase in floral organ development and male and female fertilities are revealed through characterization of GhVIN1-RNAi cotton plants. Plant Physiol. 171, 405–423 (2016). 26969720 10.1104/pp.16.00197 PMC4854712

[13] D. Sosso , Seed filling in domesticated maize and rice depends on SWEET-mediated hexose transport. Nat. Genet. 47, 1489–1493 (2015). 26523777 10.1038/ng.3422

[14] G. N. Scofield , Antisense suppression of the rice transporter gene, OsSUT1, leads to impaired grain filling and germination but does not affect photosynthesis. Funct. Plant Biol. 29, 815–826 (2002). 32689529 10.1071/PP01204

[15] Z. Chu , Promoter mutations of an essential gene for pollen development result in disease resistance in rice. Genes Dev. 20, 1250–1255 (2006). 16648463 10.1101/gad.1416306 PMC1472899

[16] T. Hirose , Disruption of a gene for rice sucrose transporter, *OsSUT1*, impairs pollen function but pollen maturation is unaffected. J. Exp. Bot. 61, 3639–3646 (2010). 20603282 10.1093/jxb/erq175 PMC2921200

[17] H. Zhang , Carbon starved anther encodes a MYB domain protein that regulates sugar partitioning required for rice pollen development. Plant Cell 22, 672–689 (2010). 20305120 10.1105/tpc.109.073668 PMC2861464

[18] L. E. Williams, R. Lemoine, N. Sauer, Sugar transporters in higher plants--A diversity of roles and complex regulation. Trends Plant Sci. 5, 283–290 (2000). 10871900 10.1016/s1360-1385(00)01681-2

[19] Q. Xu , Carbon export from leaves is controlled via ubiquitination and phosphorylation of sucrose transporter SUC2. Proc. Natl. Acad. Sci. U.S.A. 117, 6223–6230 (2020). 32123097 10.1073/pnas.1912754117 PMC7084081

[20] R. Lemoine , Source-to-sink transport of sugar and regulation by environmental factors. Front. Plant Sci 4, 272 (2013). 23898339 10.3389/fpls.2013.00272 PMC3721551

[21] K. Ljung, J. L. Nemhauser, P. Perata, New mechanistic links between sugar and hormone signalling networks. Curr. Opin. Plant Biol. 25, 130–137 (2015). 26037392 10.1016/j.pbi.2015.05.022

[22] H. Wang , The tomato Aux/IAA transcription factor IAA9 is involved in fruit development and leaf morphogenesis. Plant Cell 17, 2676–2692 (2005). 16126837 10.1105/tpc.105.033415 PMC1242265

[23] M. Sagar , SlARF4, an auxin response factor involved in the control of sugar metabolism during tomato fruit development. Plant Physiol. 161, 1362–1374 (2013). 23341361 10.1104/pp.113.213843 PMC3585602

[24] Z. Zhao , A role for a dioxygenase in auxin metabolism and reproductive development in rice. Dev. Cell 27, 113–122 (2013). 24094741 10.1016/j.devcel.2013.09.005

[25] K. I. Hayashi , The main oxidative inactivation pathway of the plant hormone auxin. Nat. Commun. 12, 6752 (2021). 34811366 10.1038/s41467-021-27020-1 PMC8608799

[26] S. Song , OsFTIP7 determines auxin-mediated anther dehiscence in rice. Nat. Plants 4, 495–504 (2018). 29915329 10.1038/s41477-018-0175-0

[27] Y. Zhao , A role for flavin monooxygenase-like enzymes in auxin biosynthesis. Science 291, 306–309 (2001). 11209081 10.1126/science.291.5502.306

[28] B. A. Ambrose , Molecular and genetic analyses of the silky1 gene reveal conservation in floral organ specification between eudicots and monocots. Mol. Cell 5, 569–579 (2000). 10882141 10.1016/s1097-2765(00)80450-5

[29] N. Nagasawa , SUPERWOMAN1 and DROOPING LEAF genes control floral organ identity in rice. Development 130, 705–718 (2003). 12506001 10.1242/dev.00294

[30] H. Yoshida, Y. Nagato, Flower development in rice. J. Exp. Bot. 62, 4719–4730 (2011). 21914655 10.1093/jxb/err272

[31] R. F. Baker , Sucrose transporter *ZmSut1* expression and localization uncover new insights into sucrose phloem loading. Plant Physiol. 172, 1876–1898 (2016). 27621426 10.1104/pp.16.00884 PMC5100798

[32] A. N. Stepanova , TAA1-mediated auxin biosynthesis is essential for hormone crosstalk and plant development. Cell 133, 177–191 (2008). 18394997 10.1016/j.cell.2008.01.047

[33] Y. Tao , Rapid synthesis of auxin via a new tryptophan-dependent pathway is required for shade avoidance in plants. Cell 133, 164–176 (2008). 18394996 10.1016/j.cell.2008.01.049 PMC2442466

[34] Y. Zhao, Essential roles of local auxin biosynthesis in plant development and in adaptation to environmental changes. Annu. Rev. Plant Biol. 69, 417–435 (2018). 29489397 10.1146/annurev-arplant-042817-040226

[35] W. M. Gray, S. Kepinski, D. Rouse, O. Leyser, M. Estelle, Auxin regulates SCF(TIR1)-dependent degradation of AUX/IAA proteins. Nature 414, 271–276 (2001). 11713520 10.1038/35104500

[36] M. Salehin, R. Bagchi, M. Estelle, SCFTIR1/AFB-based auxin perception: Mechanism and role in plant growth and development. Plant Cell 27, 9–19 (2015). 25604443 10.1105/tpc.114.133744 PMC4330579

[37] C. Shen , Functional analysis of the structural domain of ARF proteins in rice (*Oryza sativa* L.). J. Exp. Bot. 61, 3971–3981 (2010). 20693412 10.1093/jxb/erq208 PMC2935870

[38] T. Ulmasov, J. Murfett, G. Hagen, T. J. Guilfoyle, Aux/IAA proteins repress expression of reporter genes containing natural and highly active synthetic auxin response elements. Plant Cell 9, 1963–1971 (1997). 9401121 10.1105/tpc.9.11.1963 PMC157050

[39] T. J. Guilfoyle, G. Hagen, Auxin response factors. Curr. Opin. Plant Biol. 10, 453–460 (2007). 17900969 10.1016/j.pbi.2007.08.014

[40] E. J. Chapman, M. Estelle, Mechanism of auxin-regulated gene expression in plants. Annu. Rev. Genet. 43, 265–285 (2009). 19686081 10.1146/annurev-genet-102108-134148

[41] J. W. Chandler, Auxin response factors. Plant Cell Environ. 39, 1014–1028 (2016). 26487015 10.1111/pce.12662

[42] C. Grierson , Separate cis sequences and trans factors direct metabolic and developmental regulation of a potato tuber storage protein gene. Plant J. 5, 815–826 (1994). 8054988 10.1046/j.1365-313x.1994.5060815.x

[43] J. Huang, Z. Li, D. Zhao, Deregulation of the OsmiR160 target gene OsARF18 causes growth and developmental defects with an alteration of auxin signaling in rice. Sci. Rep. 6, 29938 (2016). 27444058 10.1038/srep29938 PMC4956771

[44] A. Schlereth , MONOPTEROS controls embryonic root initiation by regulating a mobile transcription factor. Nature 464, 913–916 (2010). 20220754 10.1038/nature08836

[45] P. Nagpal , Auxin response factors ARF6 and ARF8 promote jasmonic acid production and flower maturation. Development 132, 4107–4118 (2005). 16107481 10.1242/dev.01955

[46] M. M. R. Ambavaram , Coordinated regulation of photosynthesis in rice increases yield and tolerance to environmental stress. Nat. Commun. 5, 5302 (2014). 25358745 10.1038/ncomms6302 PMC4220491

[47] B. R. Shen , Engineering a new chloroplastic photorespiratory bypass to increase photosynthetic efficiency and productivity in rice. Mol. Plant 12, 199–214 (2019). 30639120 10.1016/j.molp.2018.11.013

[48] P. F. South, A. P. Cavanagh, H. W. Liu, D. R. Ort, Synthetic glycolate metabolism pathways stimulate crop growth and productivity in the field. Science 363, 45–53 (2019). 10.1126/science.aat9077 PMC7745124 30606819

[49] Y. Tan , Fast and simple droplet sampling of sap from plant tissues and capillary microextraction of soluble saccharides for picogram-scale quantitative determination with GC-MS. J. Agric. Food Chem. 58, 9931–9935 (2010). 20806935 10.1021/jf102053h

[50] F. C. Felker, D. M. Peterson, O. E. Nelson, ^[C]^Sucrose uptake and labeling of starch in developing grains of normal and segl barley. Plant Physiol. 74, 43–46 (1984). 16663383 10.1104/pp.74.1.43 PMC1066621

[51] A. Mukhopadhyay, B. Deplancke, A. J. Walhout, H. A. Tissenbaum, Chromatin immunoprecipitation (ChIP) coupled to detection by quantitative real-time PCR to study transcription factor binding to DNA in *Caenorhabditis elegans*. Nat. Protoc. 3, 698–709 (2008). 18388953 10.1038/nprot.2008.38 PMC2681100

