# Supplementary material for: Auxin regulates source-sink carbohydrate partitioning and reproductive organ development in rice
Source: Proc Natl Acad Sci U S A. 2022 Aug 29;119(36):e2121671119. doi: 10.1073/pnas.2121671119 (PMC9457257; doi:10.1073/pnas.2121671119)
Supplement: Supplementary File [file pnas.2121671119.sapp.pdf]

PNAS
www.pnas.org

Supplementary Information for
Auxin regulates source-sink carbohydrate partitioning and reproductive organ development in rice

Zhigang Zhao[a, 1, 2], Chaolong Wang[a, 1], Xiaowen Yu[a, 1], Yunlu Tian[a], Wenxin Wang[a], Yunhui Zhang[a], Wenting Bai[a], Ning Yang[c], Tao Zhang[c], Hai Zheng[a], Qiming Wang[a], Jiayu Lu[a], Dekun Lei[a], Xiaodong He[a], Keyi Chen[a], Junwen Gao[a], Xi Liu[a], Shijia Liu[a], Ling Jiang[a], Haiyang Wang[b], Jianmin Wan[a, b, 2]

To whom correspondence should be addressed. Email: wanjm@njau.edu.cn, wanjianmin@caas.cn or zhaozg@njau.edu.cn

This PDF file includes:

- Supplementary text
- Figures S1 to S14 (not allowed for Brief Reports)
- Tables S1
- Legends for Datasets S1 to S2

Other supplementary materials for this manuscript include the following:

- Datasets S1 to S2

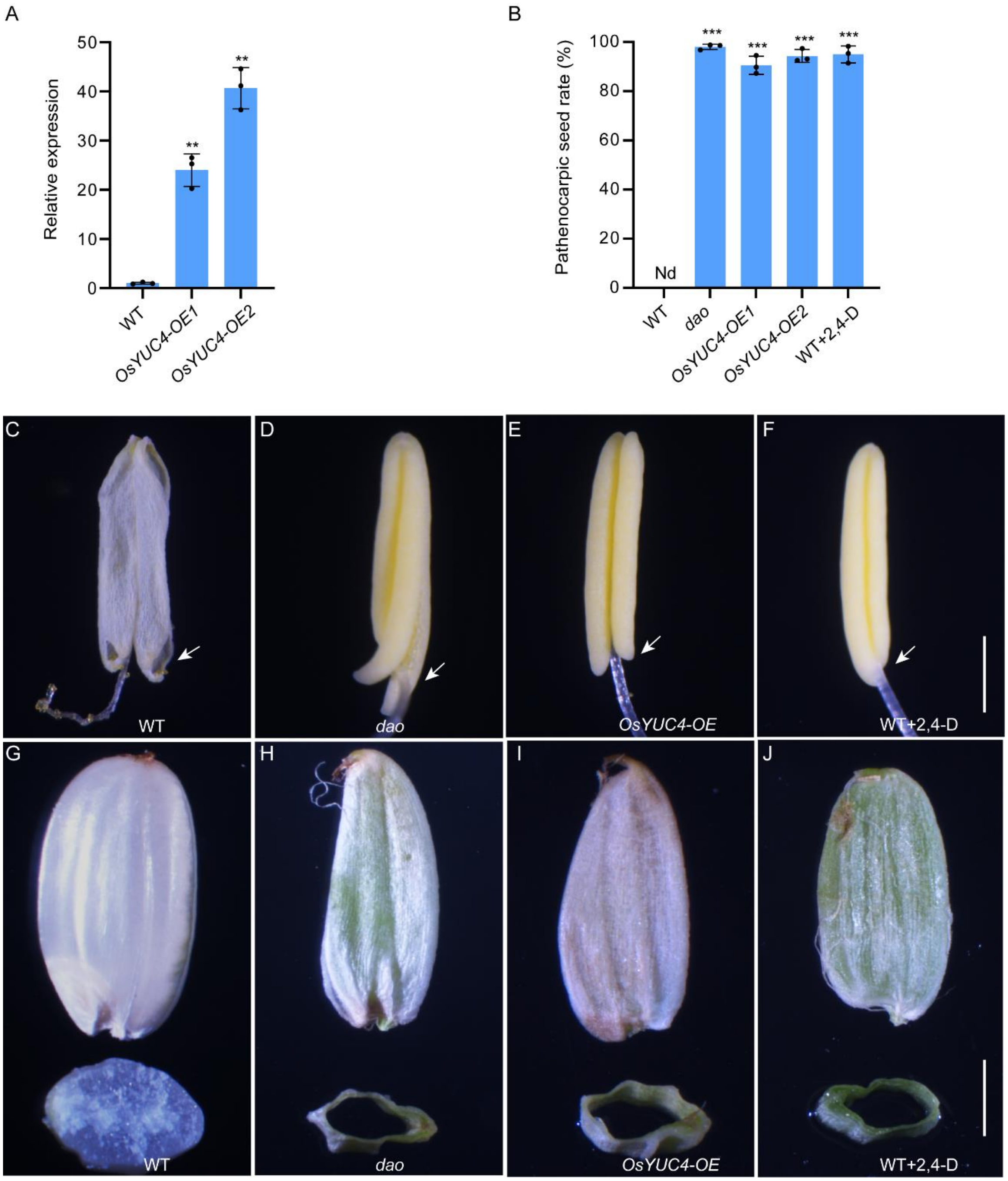

Fig. S1. qRT-PCR analysis of *OsYUC4* gene expression in the *OsYUC4-OE* transgenic plants and comparison of the anthers and seeds of WT and *dao*, *OsYUC4-OE*, and WT plants treated with 2,4-D.

(A) qRT-PCR analysis of *OsYUC4* expression in the anthers of *OsYUC4-OE* plants. Data shown are means ± SD (*n*=3; **P<0.01, based on Student's *t*-test). Ubiquitin (*LOC_Os03g13170*) was used as a control.

(B) Parthenocarpic seed rates of WT, *dao*, *OsYUC4-OE1*, *OsYUC4-OE2*, and WT plants treated with 2, 4-D (10^{-6} M) at 30 days after flowering. Data shown are means ± SD (*n*=3; ***P<0.001, based on Student's *t*-test). Nd indicates no detection.

(C-F) Comparison of anthers of WT (C), *dao* (D), *OsYUC4-OE* (E), and WT plants treated with exogenous 2,4-D (10^{-6} M) (F). Arrows indicate anther dehiscence. Bar=2 mm.

(G-J) Comparison of seeds of WT (G), *dao* (H), *OsYUC4-OE* (I), and WT plants treated with exogenous 2,4-D (10^{-6} M) (J). Bar=1 mm.

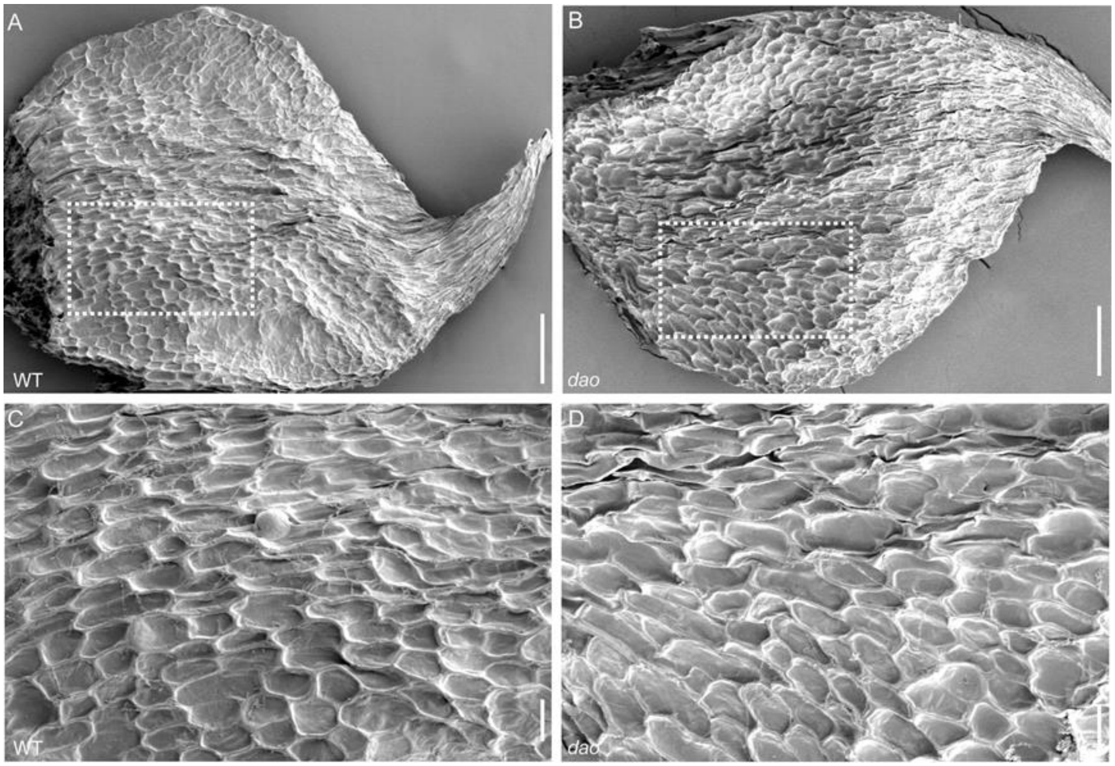

Fig. S2. Scanning electron microscopy images of the lodicules in WT (A) and *dao* mutants (B). Bar=300 µm. (C) and (D) are the enlarged images of the dotted box in (A) and (B), respectively. The cell wall structure of lodicule of the *dao* mutant is apparently normal compared to that of the WT lodicule. Bar=500 µm.

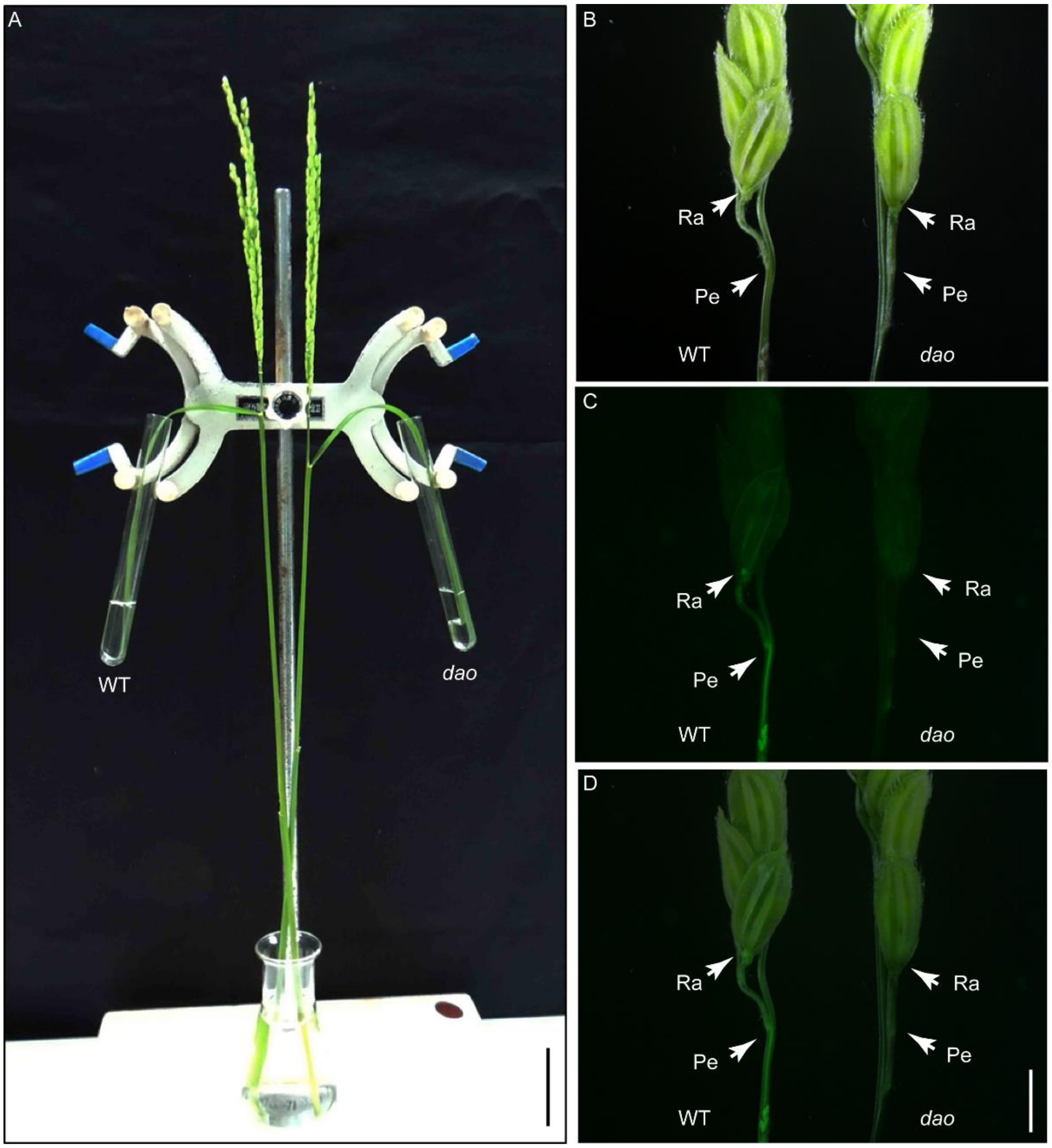

Fig. S3. The experimental design of sucrose translocation from source (flag leaves) to sink (panicles).

(A) The experimental design of sucrose translocation. Bar=5 cm.

(B-D) 5, 6-CFDA fluorescence accumulation in the panicles of WT and *dao* mutants. The images were taken in the bright-field (B), in the fluorescence (C); or merged (D) of (B) and (C). Arrows indicate the fluorescence in the rachilla and pedicel of the panicles. Ra rachilla, Pe pedicel. Bar=10 cm.

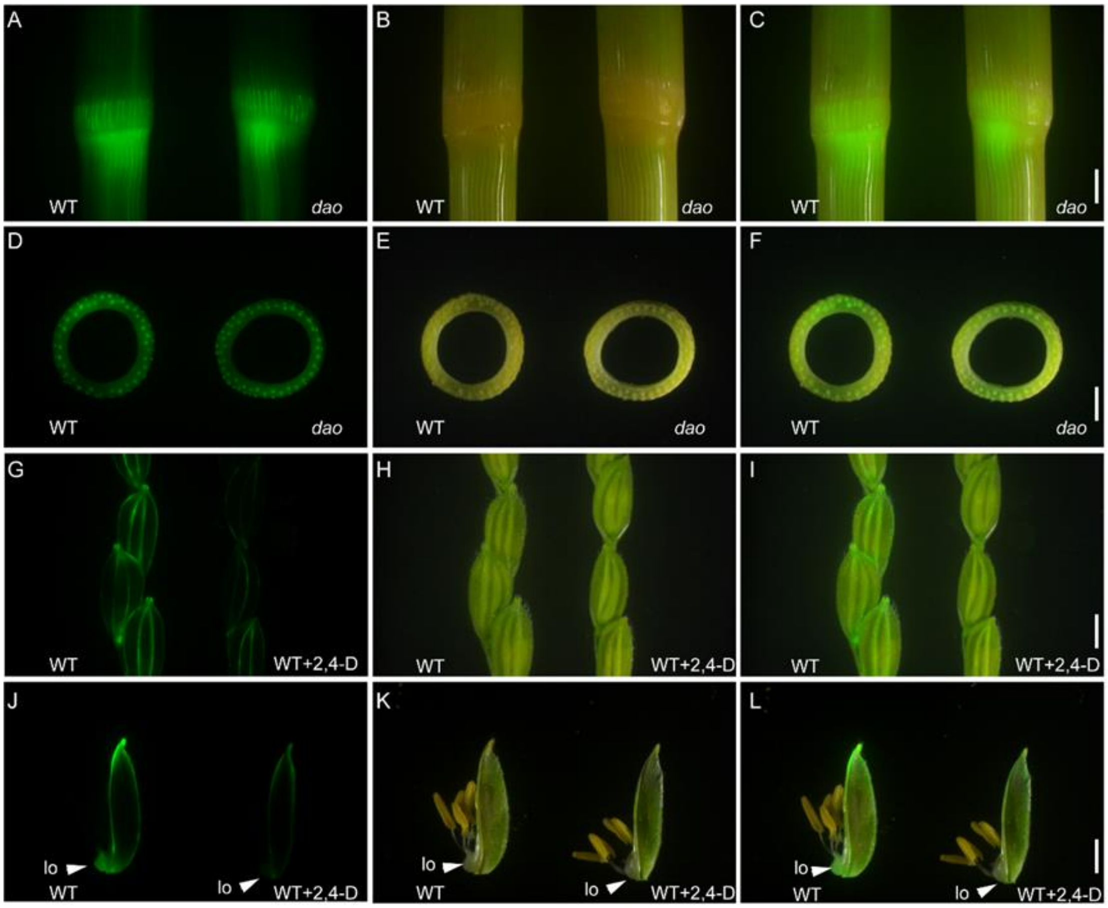

Fig. S4. The sucrose translocation from source to sink.

(A-C) 5, 6-CFDA fluorescence accumulation in the stems of WT and *dao* mutant. The stems are shown in the fluorescence (A), and in the bright-field (B); and in the merged image (C). (D-F) 5, 6-CFDA fluorescence accumulation in the cross-sections of stems. The cross-sections of stems are shown in the fluorescence (D), and in the bright-field (E); and in the merged image (F). (G-I) 5, 6-CFDA fluorescence accumulation in the panicles of WT plants treated with 2, 4-D (10^{-6} M). The panicles are shown in the fluorescence (G), and in the bright-field (H); and in the merged image (I). (J-L) 5, 6-CFDA fluorescence accumulation in the lodicules of WT and WT plants treated with 2, 4-D (10^{-6} M) (auxin applied to the flag leaf). The lodicules are shown in the fluorescence (J), and in the bright-field (K); and in the merged image (L). Arrows indicate the lodicule (Lo). Bar=10 cm.

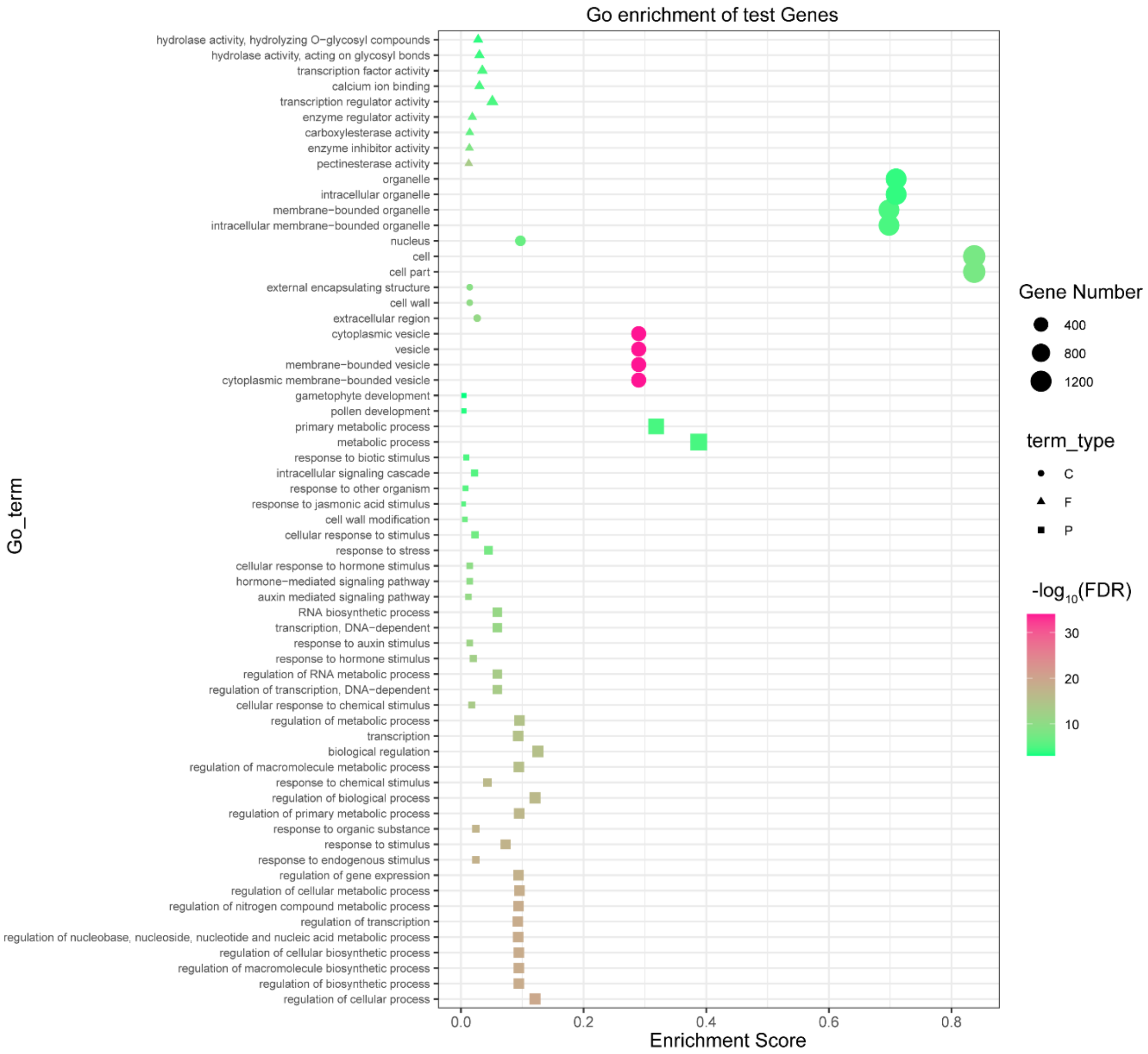

Fig. S5. GO term analysis of the biological processes enriched in the DEGs of WT and *dao* lodicules.

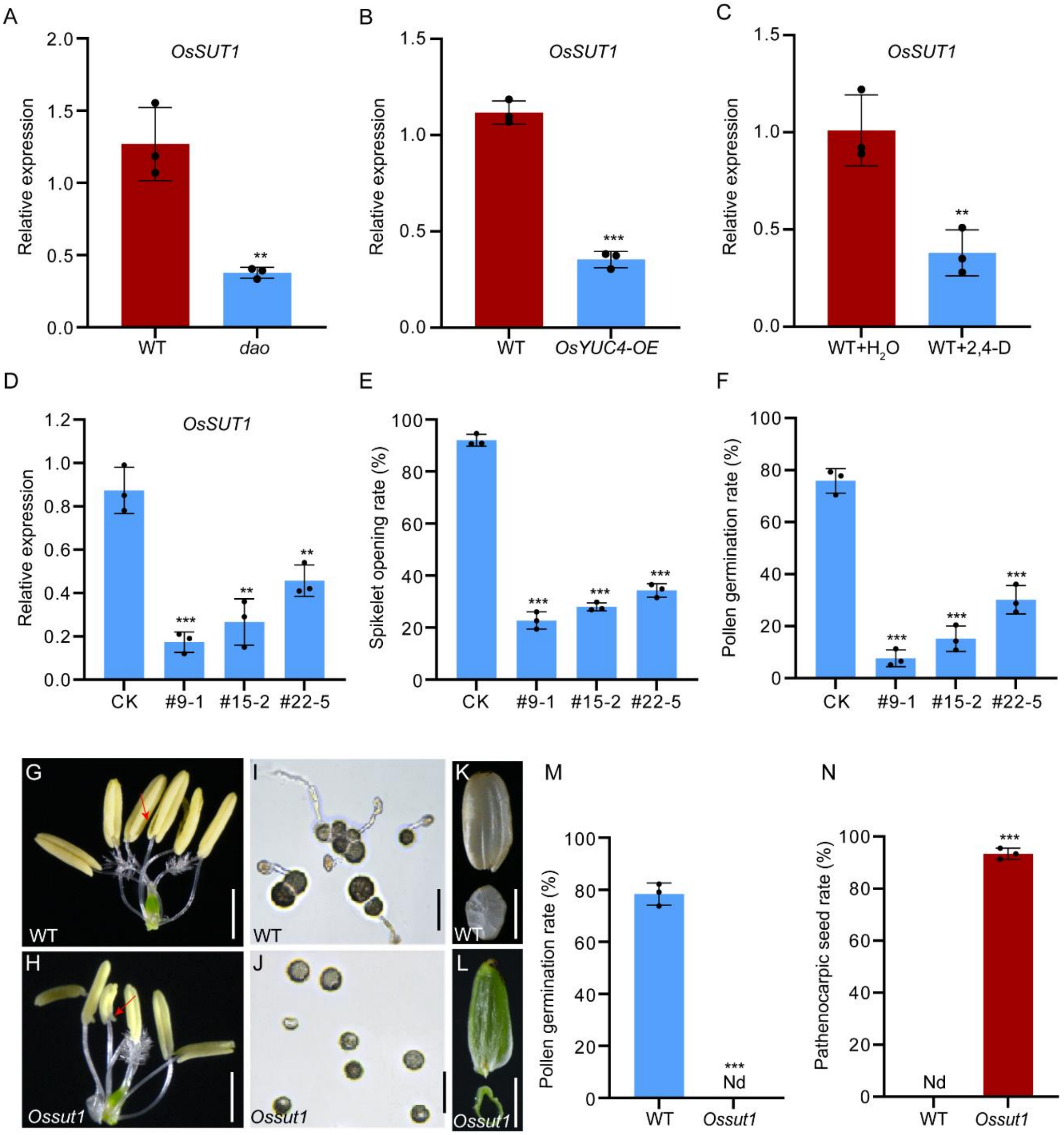

Fig. S6. Expression analysis of *OsSUT1* and phenotypic comparison of WT and the *Ossut1* mutant.

(A-C) qRT-PCR analysis of *OsSUT1* expression in the flag leaves of WT and *dao* mutant (A), *OsYUC4-OE* (B), and WT plants treated with 2,4-D and water (control) (C), respectively. Data are shown as means ± SD (*n*=3; ***P*<0.01, ****P*<0.001, based on Student's *t*-test).

(D) Relative expression of *OsSUT1* in the *OsSUT1* RNAi transgenic plants and WT plants (CK). Ubiquitin (*LOC_Os03g13170*) was used as a control. Data shown are means ± SD (n=3; **P<0.01, ***P<0.001, based on Student's t-test).
(E and F) Spikelet opening rate (E) and pollen germination rate (F) of the *OsSUT1* RNAi transgenic plants. Data shown are means ± SD (n=3; **P<0.01, ***P<0.001, based on Student's t-test).
(G-I) Comparison of the anthers, germinating pollen and seeds of WT (G, I and K) and *Ossut1* mutant plants (H, J and L).
(M) In vitro germination assay shows that the pollen of *Ossut1* mutants could not normally germinate compared to that of WT. Data shown are means ± SD (n=3). Nd indicates no detection.
(N) High parthenocarpic seed rate in the *Ossut1* mutant. Data shown are means ± SD (n=3; ***P<0.001, based on Student's t-test). Nd indicates no detection.

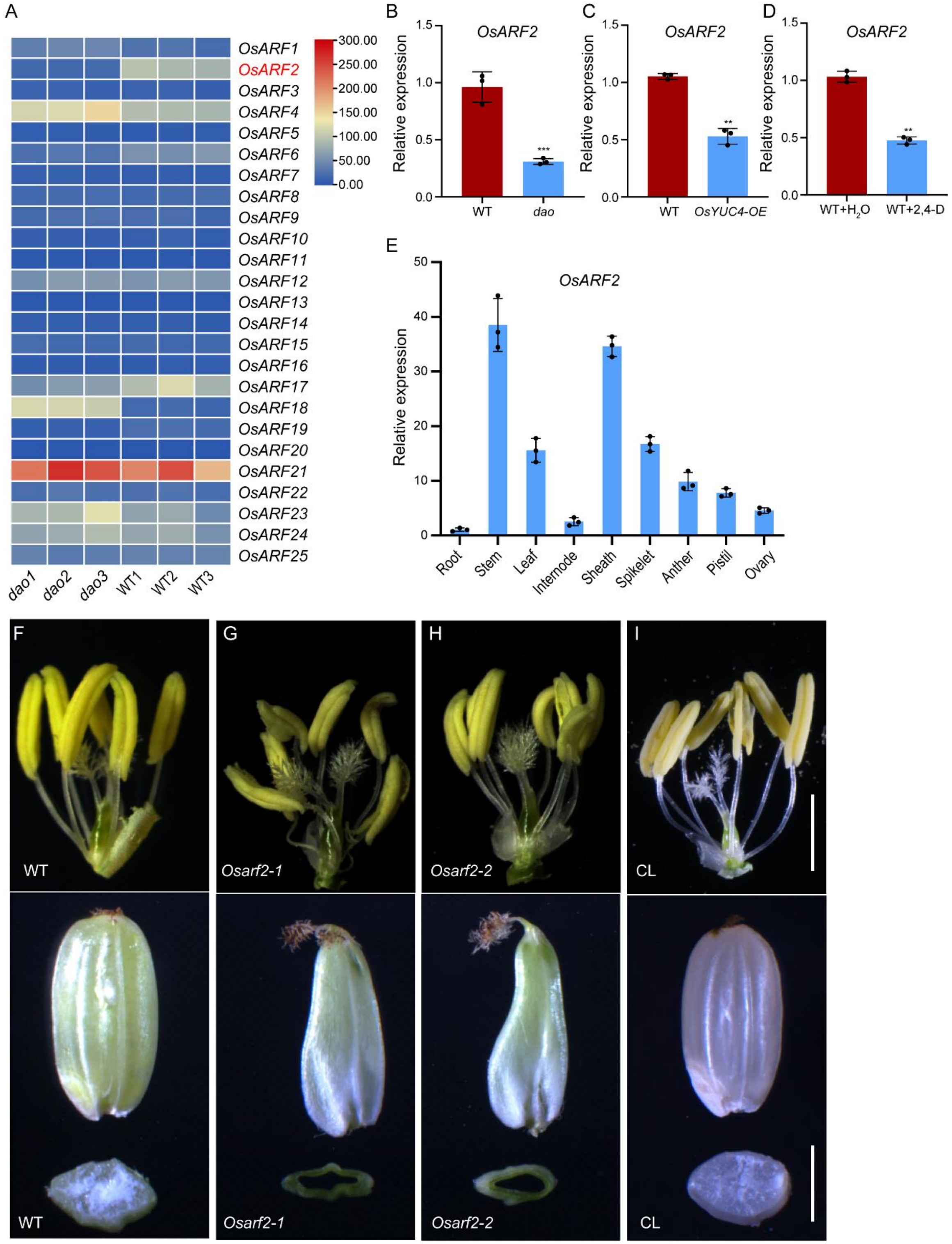

Fig. S7. Expression and functional analysis of the *OsARF2* gene.

(A) Expression heat map of the different *OsARFs* genes in WT and the *dao* mutant. The color key (red to blue) represents gene expression FPKM as fold change.
(B-D) qRT-PCR analysis of *OsARF2* expression in the flag leaves of WT and *dao* mutant (B), *OsYUC4-OE* (C), and WT plants treated with 2,4-D and water (control) (D), respectively. Data shown are means ± SD (*n*=3; ***P*<0.01, ****P*<0.001, based on Student's *t*-test).
(E) qRT-PCR analysis showing higher expression of *OsARF2* in the mature stem, sheath and spikelet. Values are means ± SD (*n*=3 biological replicates). Ubiquitin (*LOC_Os03g13170*) was used as a control.
(F-I) Comparison of the spikelets, anthers, and seeds of WT (F), *Osarf2-1* (G), *Osarf2-2* (H) and the complemented line of *Osarf2-1* (I). Bar=5 mm.

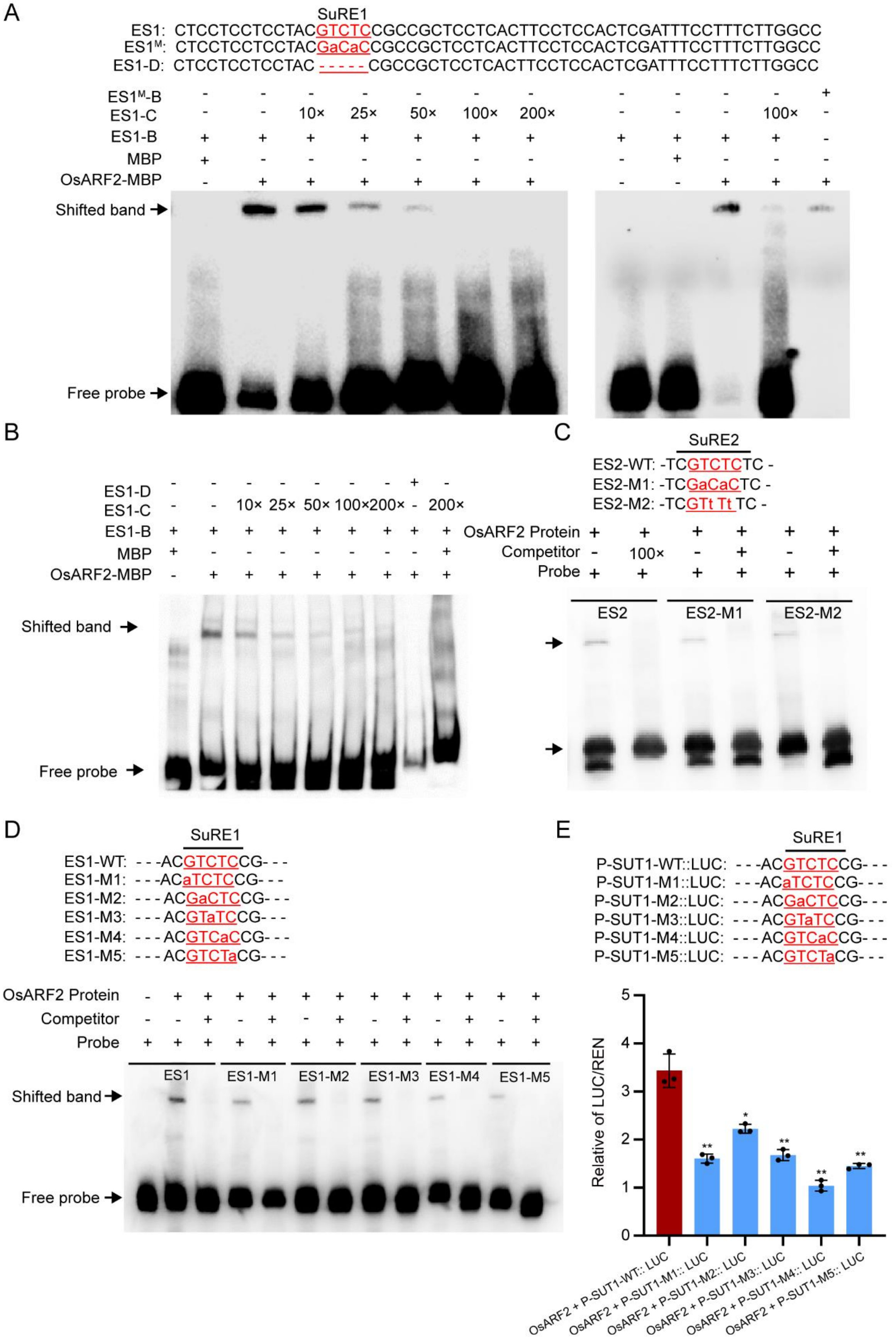
A
SuRE1
ES1: CTCCTCCTCCTACGTCTCCGCCGCTCCTCACTTCCTCCACTCGATTTCCTTTCTTGGCC
ES1M: CTCCTCCTCCTACGaCaCCGCCGCTCCTCACTTCCTCCACTCGATTTCCTTTCTTGGCC
ES1-D: CTCCTCCTCCTAC - - - - - CGCCGCTCCTCACTTCCTCCACTCGATTTCCTTTCTTGGCC
ES1M-B
ES1-C
ES1-B
MBP
OsARF2-MBP
10× 25× 50× 100× 200× 100×
Shifted band
Free probe
B
ES1-D
ES1-C
ES1-B
MBP
OsARF2-MBP
10× 25× 50× 100× 200× 200×
Shifted band
Free probe
C
SuRE2
ES2-WT: -TCGTCTCTC -
ES2-M1: -TCGaCaCTC -
ES2-M2: -TCGTt Tt TC -
OsARF2 Protein
Competitor
Probe
100×
ES2
ES2-M1
ES2-M2
D
SuRE1
ES1-WT: - - -ACGTCTCCG- - -
ES1-M1: - - -ACaTCTCCG- - -
ES1-M2: - - - ACGaCTCCG- - -
ES1-M3: - - - ACGTaTCCG- - -
ES1-M4: - - - ACGTCaCCG- - -
ES1-M5: - - - ACGTCTaCG- - -
OsARF2 Protein
Competitor
Probe
ES1
ES1-M1
ES1-M2
ES1-M3
ES1-M4
ES1-M5
Shifted band
Free probe
E
SuRE1
P-SUT1-WT::LUC: - - -ACGTCTCCG- - -
P-SUT1-M1::LUC: - - -ACaTCTCCG- - -
P-SUT1-M2::LUC: - - - ACGaCTCCG- - -
P-SUT1-M3::LUC: - - - ACGTaTCCG- - -
P-SUT1-M4::LUC: - - - ACGTCaCCG- - -
P-SUT1-M5::LUC: - - - ACGTCTaCG- - -
Relative of LUC/REN
0
1
2
3
4
5
OsARF2 + P-SUT1-WT:: LUC
OsARF2 + P-SUT1-M1:: LUC
OsARF2 + P-SUT1-M2:: LUC
OsARF2 + P-SUT1-M3:: LUC
OsARF2 + P-SUT1-M4:: LUC
OsARF2 + P-SUT1-M5:: LUC

Fig. S8. EMSA showing the binding of OsARF2 to the SuRE element in the *OsSUT1* promoter.

(A) EMSA showing OsARF2 binding to the SuRE elements in the *OsSUT1* promoter. Upper panel, the oligonucleotide sequences of the *OsSUT1* promoter probes and mutated SuRE probes used in EMSA. Underlined letters indicate the sequences of the SuRE1 motifs (GTCTC). Mutated probes are indicated by lower-case letters. For the competition test, non-labeled probes of different concentrations (from 10 to 200 x) are indicated by probe-ES1-C, labeled probes are indicated by probe-ES-1-B, and mutated probes are indicated by probe-ES-1^{M}-B, respectively.

(B) Deleted bases are indicated by dashed lines. For the competition test, non-labeled probe with different concentrations (from 10 to 200 times) are used.

(C) EMSA for mutations in the SuRE2 motif in the *OsSUT1* promoter.

(D) EMSA for single-nucleotide mutations of the five nucleotides in the SuRE1 motif in the *OsSUT1* promoter.

(E) Effects of OsARF2 co-expression on luciferase expression driver by single-nucleotide mutation of the five nucleotides in the SuRE1 motif in the *OsSUT1* promoter. Values are means ± SD (n=3 biological replicates). Significance analysis was conducted with the Student's *t*-tests (*P<0.05, **P<0.01, based on Student's *t*-test).

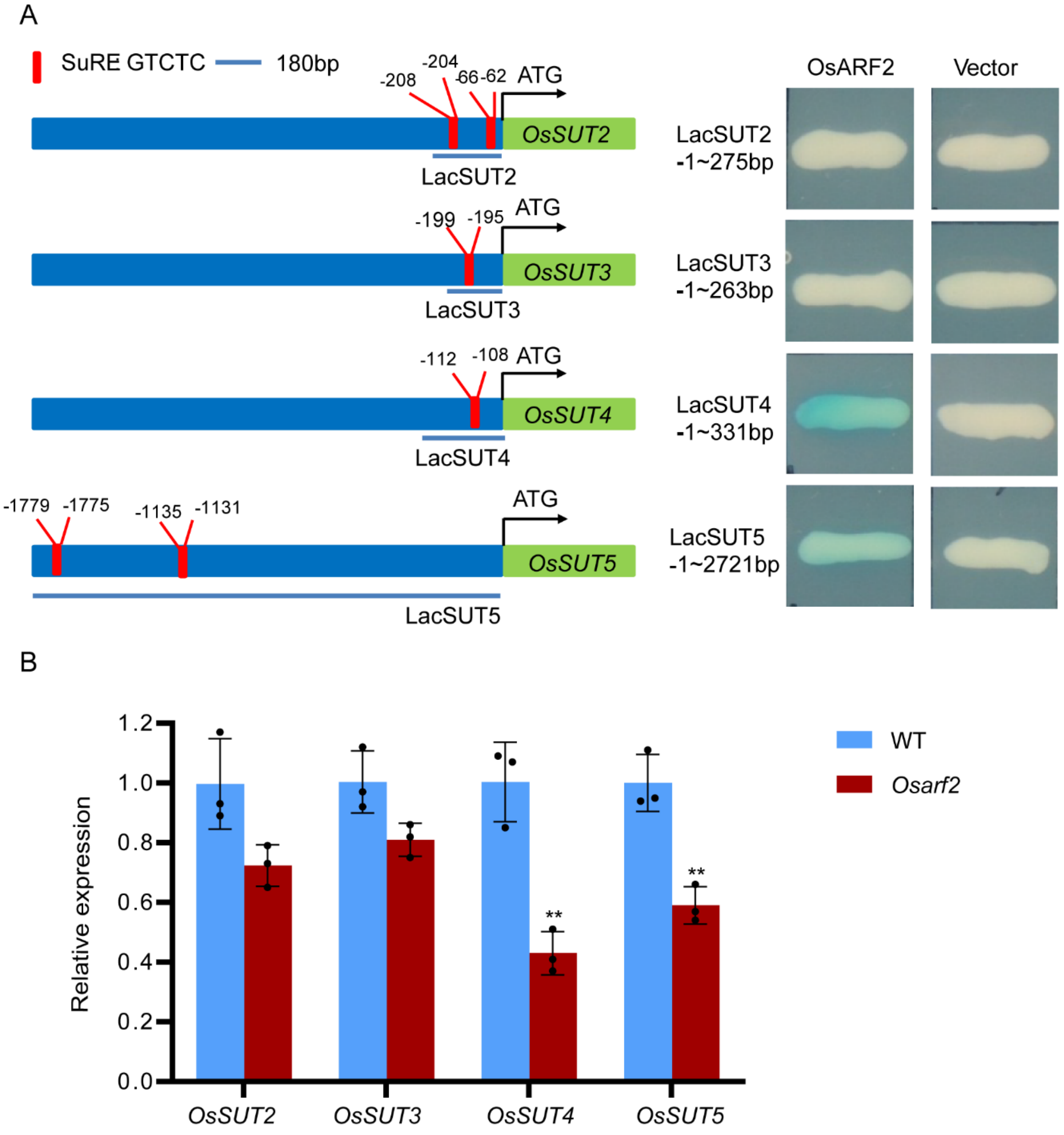

Fig. S9. OsARF2 regulates the expression of several sucrose transporter genes. (A) Yeast one-hybrid assay shows that OsARF2 could bind to the promoters of *OsSUT4* and *OsSUT5*.

(B) qRT-PCR analysis of the expression of *OsSUT2*, *OsSUT3*, *OsSUT4* and *OsSUT5* in the *Osarf2* mutant. Data shown are means ± SD (*n*=3; **P<0.01, based on Student's *t*-test).

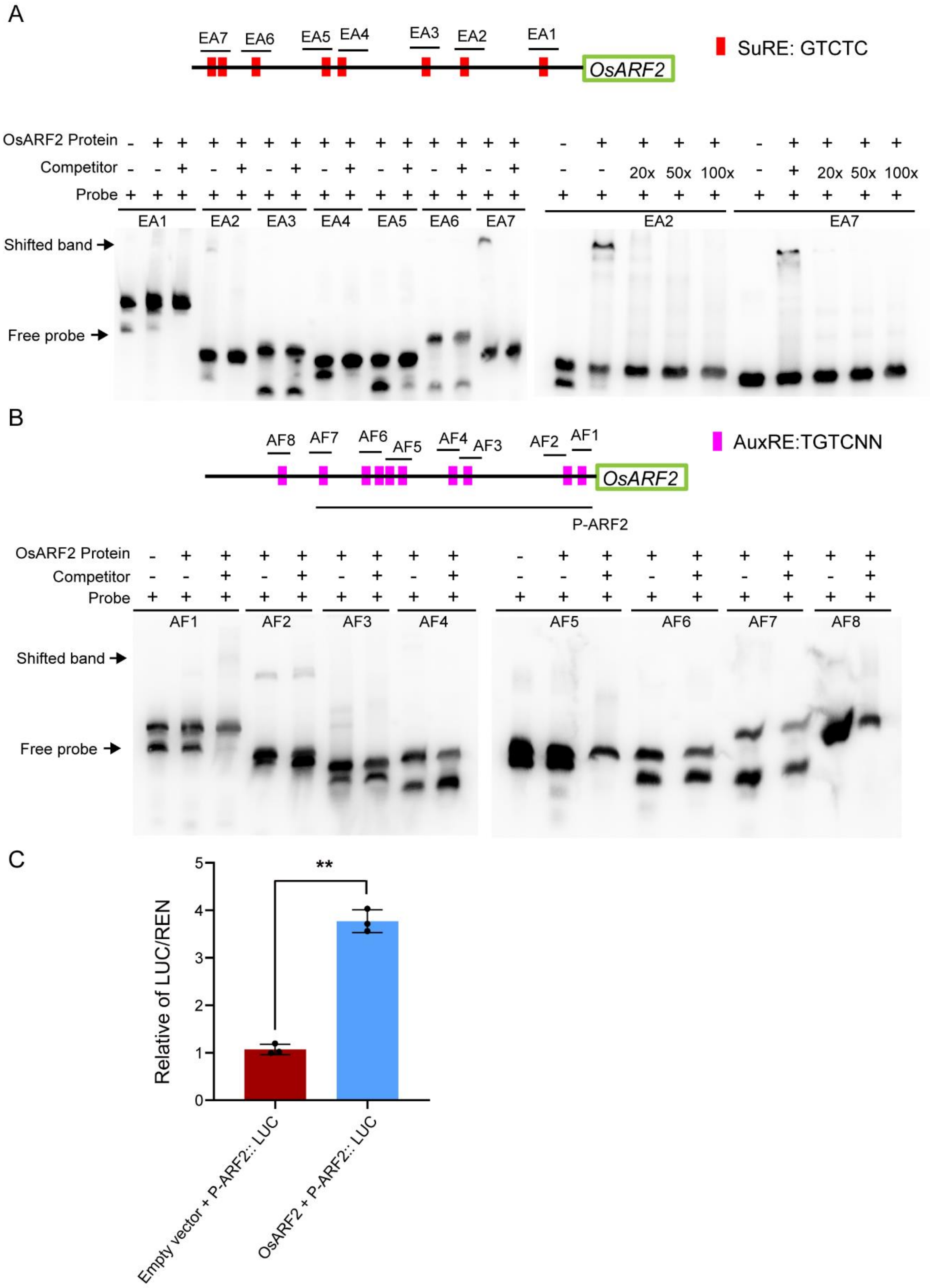
A
EA7
EA6
EA5
EA4
EA3
EA2
EA1
OsARF2
SuRE: GTCTC
OsARF2 Protein
Competitor
Probe
EA1 EA2 EA3 EA4 EA5 EA6 EA7
20x 50x 100x
EA2
EA7
Shifted band
Free probe
B
AF8 AF7 AF6 AF5 AF4 AF3 AF2 AF1
OsARF2
AuxRE:TGTCNN
P-ARF2
OsARF2 Protein
Competitor
Probe
AF1 AF2 AF3 AF4 AF5 AF6 AF7 AF8
Shifted band
Free probe
C
**
Relative of LUC/REN
0
1
2
3
4
5
Empty vector + P-ARF2:: LUC
OsARF2 + P-ARF2:: LUC

Fig. S10. EMSA of OsARF2 binding to the SuRE elements in its own promoter.
(A) EMSA showing direct binding of OsARF2 to the EA2 and EA7 fragments (containing SuRE elements) in the *OsARF2* promoter. Competition test of the binding specificity of OsARF2 to the EA2 and EA7 fragments.
(B) A series of probes for the OsARF2 promoter were designed and EMSA assay showed that the probes containing AuxRE elements were not bound by OsARF2.
(C) Luciferase reporter gene assay showed that OsARF2 could positively regulate its own expression. Values are means ± SD (n=3 biological replicates). Significance analysis was conducted with the Student's t-tests (**P<0.01, based on Student's t-test).

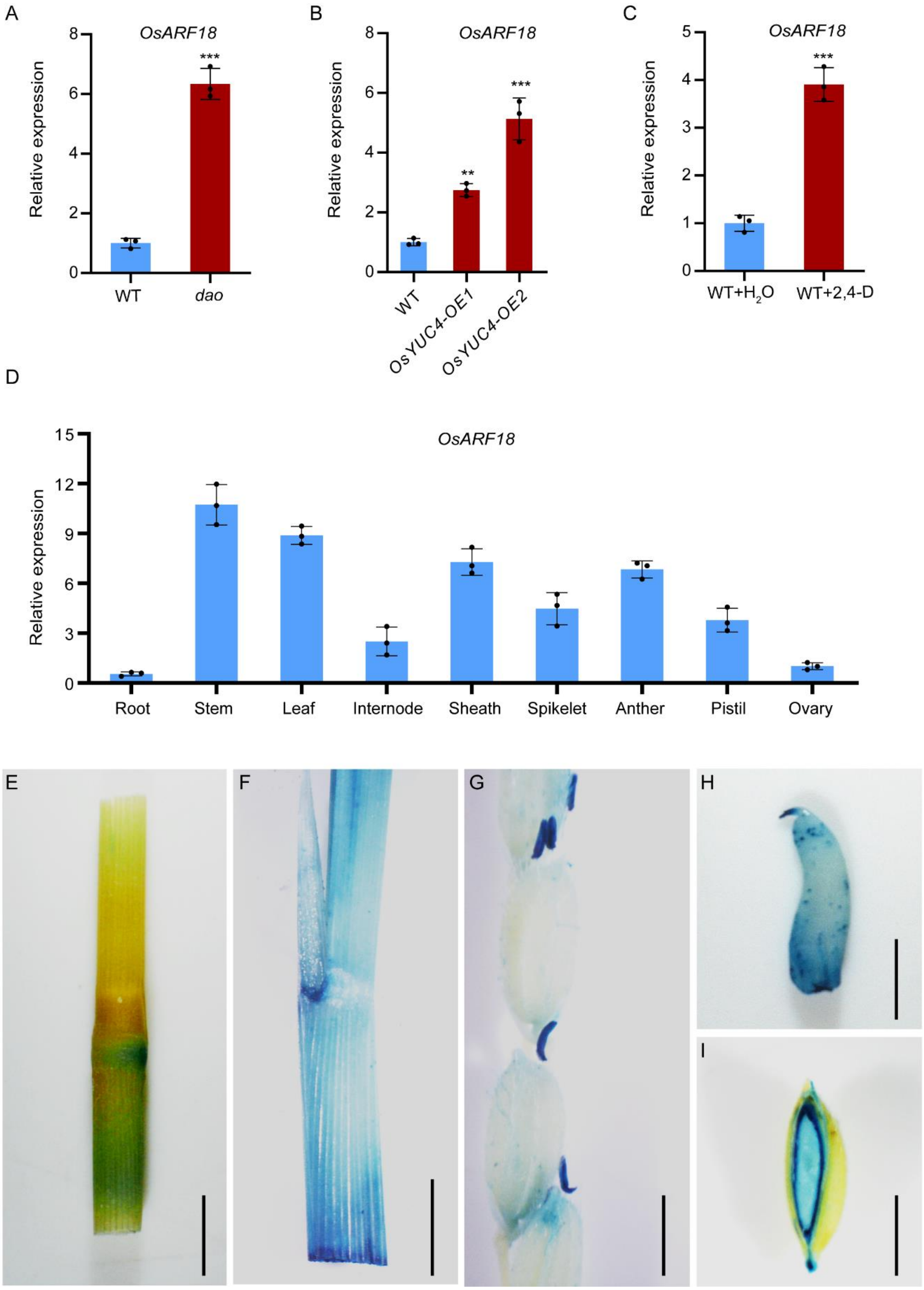

Fig. S11. Expression pattern of the *OsARF18* gene.

(A-C) qRT-PCR analysis of *OsARF18* in the *dao* mutant (A), *OsYUC4-OE1* and *OsYUC4-OE2* lines (B), and WT plants treated with 2,4-D (10^{-6} M) (C). Data shown are means ± SD (n=3; **P<0.01, ***P<0.001, based on Student's t-test).

(D) Spatial and temporal expression analysis of *OsARF18* by qRT-PCR.

(E-I) *OsARF18::GUS* reporter gene expression pattern. *GUS* expression was observed in the stem (E), sheath (F), anther (G), and fertilized ovary (H and I). Bar= 1 cm.

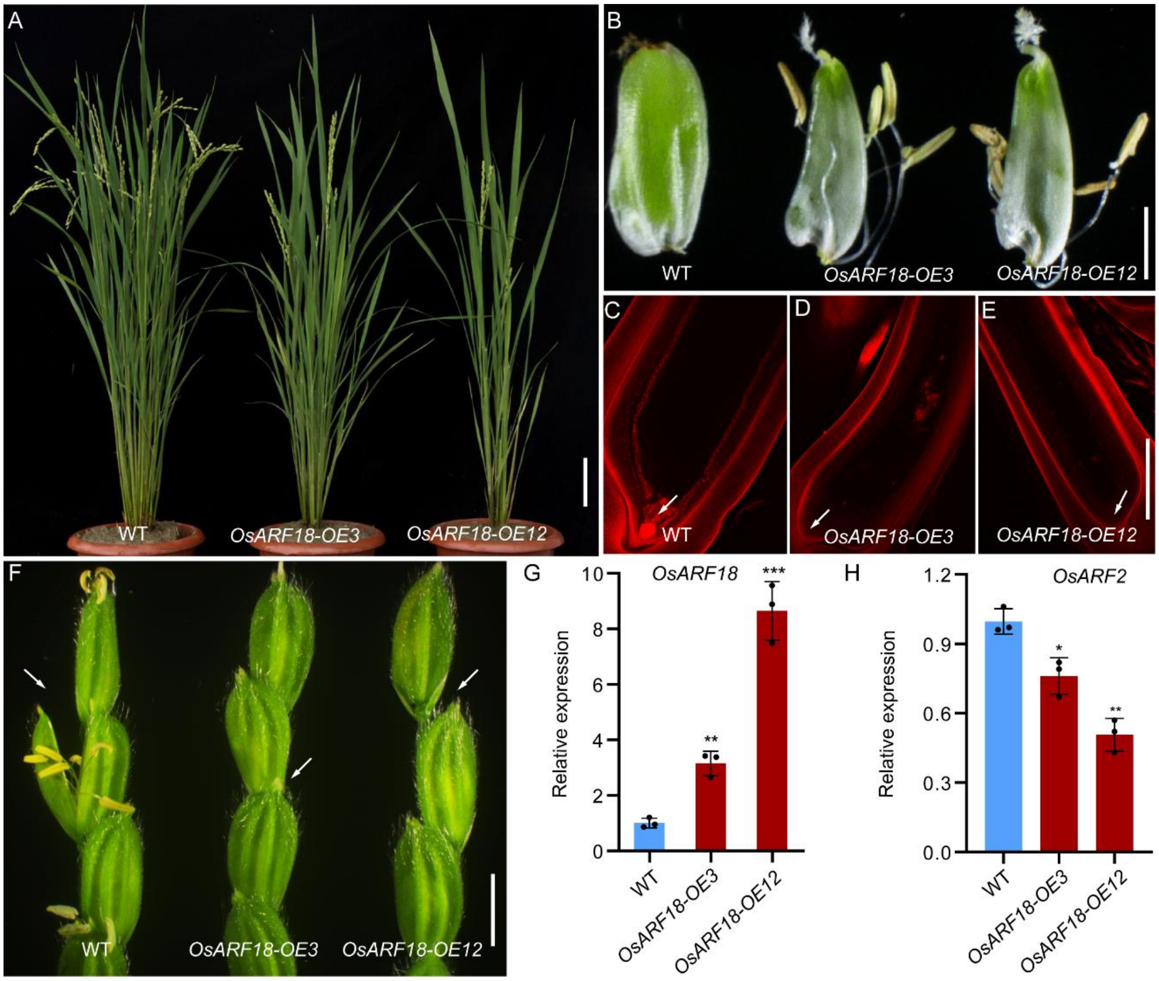

Fig. S12. Generation and phenotypic analysis of the *OsARF18-OE* transgenic plants.

(A) Phenotype of a WT plant (left) and *OsARF18-OE* plant (right) at the mature stage. Bar=10 cm.

(B) Comparison of seed morphology of WT, *OsARF18-OE3* and *OsARF18-OE12* plants. Bar=5 cm.

(C-E) Comparison of WT (C), *OsARF18-OE3* (D) and *OsARF18-OE12* (E) embryos and endosperms. Arrows indicate the embryo. Bar=50μm.

(F) Comparison of WT, *OsARF18-OE3* and *OsARF18-OE12* panicles at the heading stage. Arrows indicate spikelet opening. Bar=10 mm.

(G) qRT-PCR analysis of *OsARF18* expression in the anthers of *OsARF18-OE* transgenic plants. Data shown are means ±SD (n=3; **P<0.01, ***P<0.001, based on Student's t-test). Ubiquitin (*LOC_Os03g13170*) was used as a control.

(H) qRT-PCR analysis of *OsARF2* expression in anthers of WT and *OsARF18-OE* plants. Data shown are means ± SD (n=3; *P<0.05, **P<0.01, based on Student's t-test). Ubiquitin (*LOC_Os03g13170*) was used as a control.

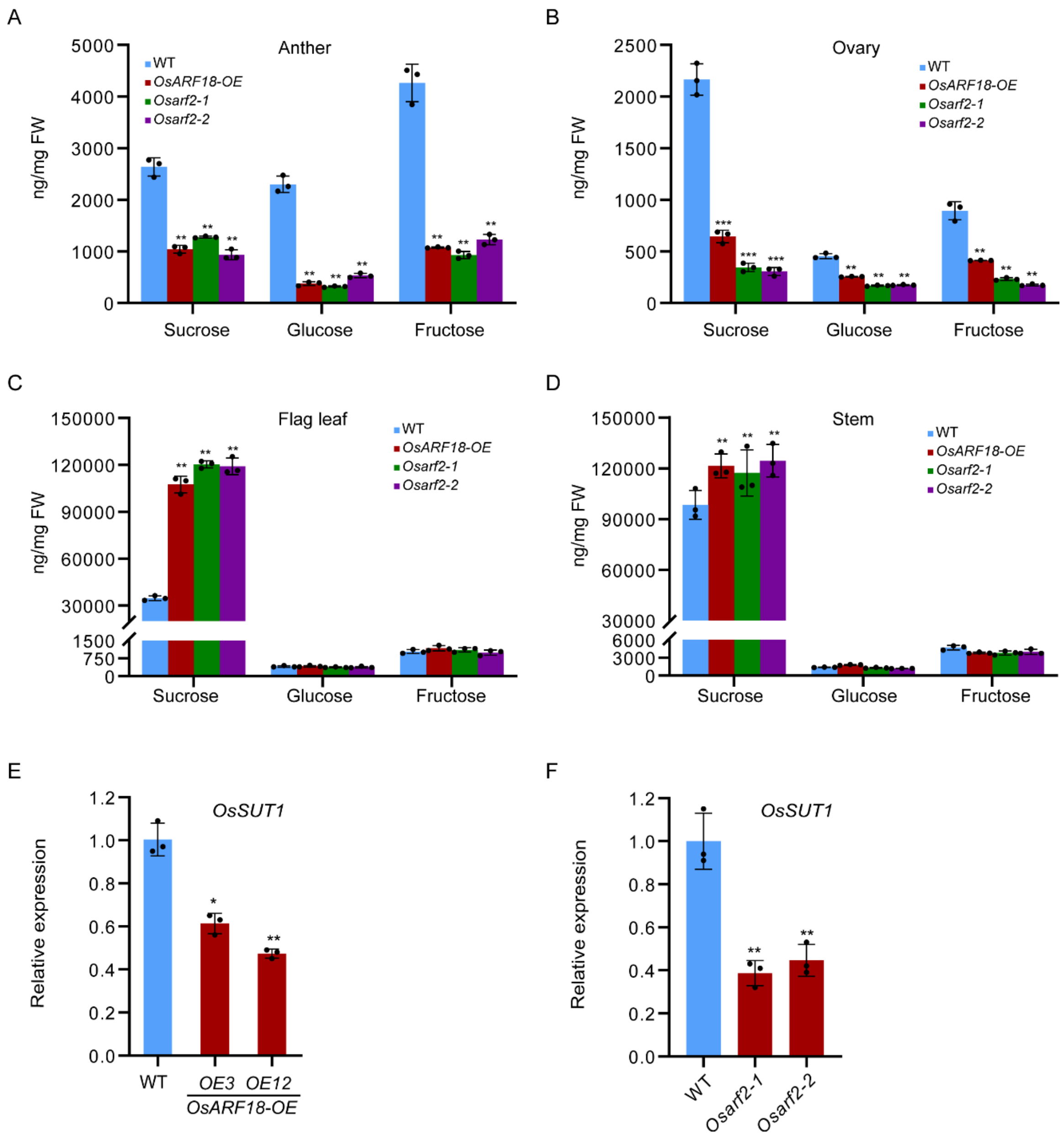

Fig. S13. The expression of *OsSUT1* and sugar levels in the WT, *OsARF18-OE* and *Osarf2* mutant plants.

(A-D) Sugar levels in anthers (A), ovaries (B), flag leaves (C), and stems (D) of WT, *OsARF18-OE* lines and *Osarf2* mutants. Data shown are means ± SD (*n*=3; **P*<0.05, ***P*<0.01, ****P*<0.001, based on Student's *t*-test).

(E and F) qRT-PCR analysis of *OsSUT1* expression in the WT and *OsARF18-OE* plants (E), and *Osarf2* mutants (F). Data shown are means ± SD (*n*=3; **P*<0.05,

**P<0.01, based on Student's *t*-test). Ubiquitin (*LOC_Os03g13170*) was used as a control.

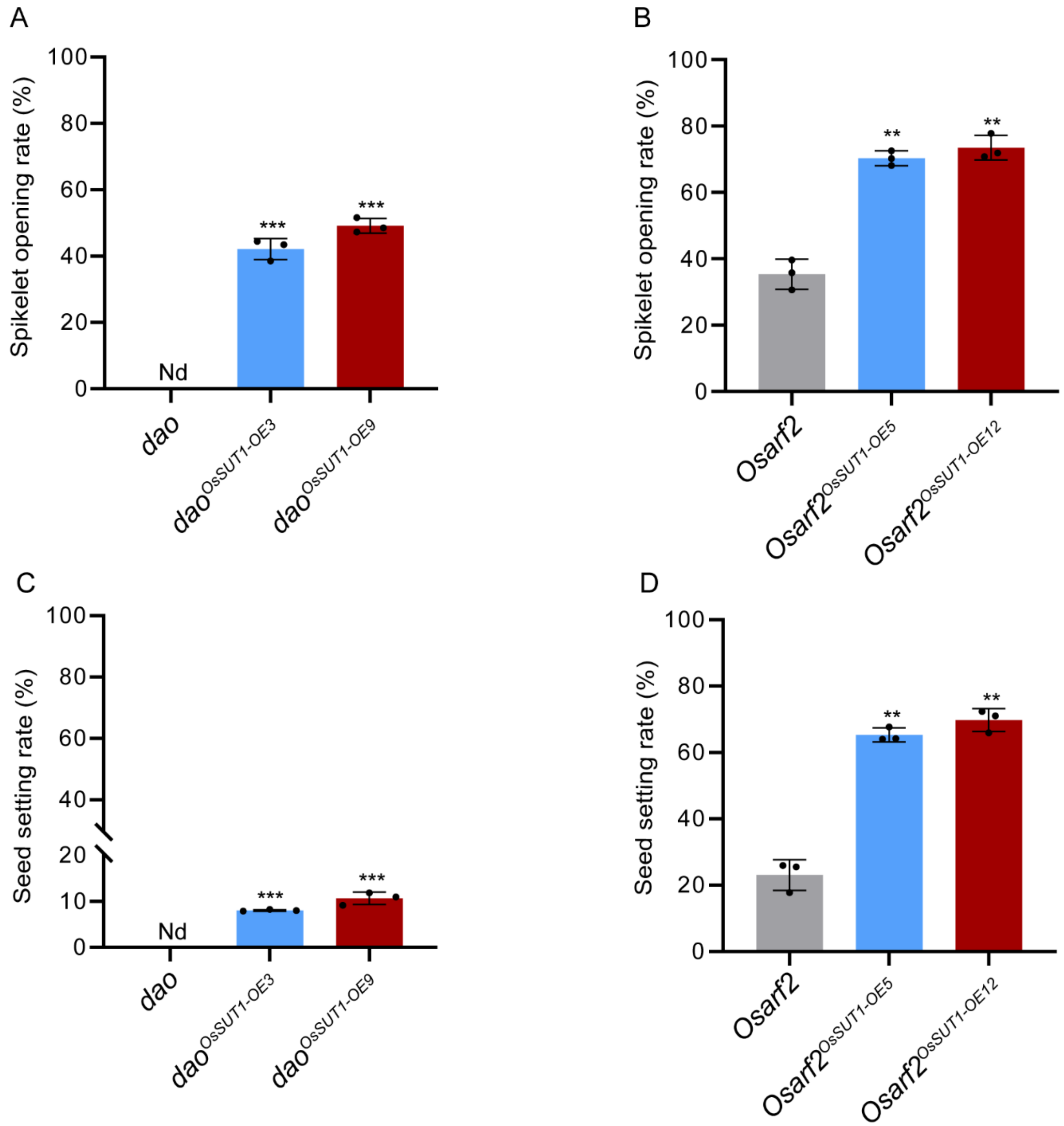

Fig. S14. Comparison of the spikelet opening rate and seed setting rate of *dao*, *Osarf2* mutants and *OsSUT1-OE* plants.

(A and B) Overexpression of *OsSUT1* in the *dao* (A) and *Osarf2* (B) background significantly increases their spikelet opening rate.

(C and D) Overexpression of *OsSUT1* in the *dao* (C) and *Osarf2* (D) background significantly increases their seed setting rate. Data shown are means ± SD (n=3). Nd indicates not detected. Data shown are means ± SD (n=3; **P<0.01, ***P<0.001, based on Student’s t-test).

Tables S1. Primers used for plasmid construction and functional analysis.

Primer name	Primer sequence (5'-3')	Description
Primers used in transgenic construction.		
OsARF2-geno-F	5'-CCGGCGCGCCAAGCTTTGCTACATGTAGCATGGTGTTTCA-3'	Complement construct
OsARF2-geno-R	5'-GAATTCCCGGGGATCCTAAAGACCGACCGAGACACACACAC-3'	
OsSUT1-RNAi-F	5'-TTCTGCACTAGGTACCCCGATCAGCCTCGGGAGGCTCA-3'	RNAi construct1
OsSUT1-RNAi-R	5'-CTGACGTAGGGGCGATAGAGCTCTCCAAGAACAGAAGATGGAGTTAG-3'	
OsARF2-pro-GUS-F	5'-GAATTCCCGGGGATCCGGGAAGATACGCAAAATGAGG -3'	Promoter for GUS analysis
OsARF2-pro-GUS-R	5'-GGCCAGTGCCAAGCTTCCGCCTCTCCACTTCCTGAAA-3'	
pGEX-OsARF18-F	5'-TGGATCCCCGGAATTCATGATAACTTTCGTGGATTCC-3'	GST fusion OsARF18 protein construct
pGEX-OsARF18-R	5'-GGCCGCTCGAGTCGACCTACCTCGCTAAGTTATCGCC -3'	
1305-OsARF2-F	5'-CGGAGCTAGCTCTAGAATGCGGGAGGGAGAGGACGGC-3'	GST fusion OsARF2 protein construct
1305-OsARF2-R	5'-TGCTCACCATGGATCCGATCATCGTATTCACTGCTGA -3'	
OsYUCCA4-OE-F	5'-GCAGGTCGACGGATCCATGGACTGCTTCGCGGAGACGGAG-3	*OsYUCCA4* overexpress construct
OsYUCCA4-OE-R	5'-GAATTCCCGGGGATCCTCAGTTGTTCTCACTTCTTTCATA-3'	
OsARF1-F	5'–CCCCAAGAGATCCAAAGCCATC-3'	Real time-PCR
OsARF1-R	5'- GCTAGATCCACAGCTCTGCCCA-3'	
OsARF2-F	5'-AAGAAGGCGTACTGGAATAA-3'	
OsARF2-R	5'-ATCCCATTTCACCACTAAAC-3'	
OsARF3-F	5'-AATGAAGCGATTTTCAAAGC-3'	
OsARF3-R	5'-CGTACTCAGAAGCAGTAGCC-3'	
OsARF4-F	5'- CTGCTTTGCCGTGTCCTCAAC-3'	
OsARF4-R	5'-CTTCTCCACCGCCATCTCATT-3'	
OsARF5-F	5'- GATCACCATCCAAGCTCATCT-3'	

OsARF5-R	5'-GCTGGAAACTGGTCTACAAGG-3'	
OsARF6-F	5'-TTCCTCCGCTGGATTTCACTC-3'	
OsARF6-R	5'-TACAAAGACGCTCCAACCCGT-3'	
OsARF7-F	5'- GGTCAATGTAGAACTTAGGGC-3'	
OsARF7-R	5'-ATGTGGCTCAGGTTTTGGGCT-3'	
OsARF8-F	5'-GAGATGTTCTGCATCGACAG-3'	
OsARF8-R	5'-TCGGTTAGTATGGTAAGCCT-3'	
OsARF9-F	5'- GGCGGTTTCTCTGTTCTTAGGA-3'	
OsARF9-R	5'-AGATGTGACGGAAATGCCACTC-3'	
OsARF10-F	5'-GGCGGGAGGAGGAGAATAGTAG-3'	
OsARF10-R	5'-CTGTAGTCCAGCTCCGGGAAGA-3'	
OsARF11-F	5'- TCTCAGTTGCTGTGTCAAGTTC-3'	
OsARF11-R	5'- TGGAATAGGGAATACATCCGTT-3'	
OsARF12-F	5'-GACGAGGTGTACGCGCAGATGA-3'	
OsARF12-R	5'- GTGTCGCTCGCCGTCAATGTCT -3'	
OsARF13-F	5'- GATCTTCAGATTTTAGTTCCCC-3'	
OsARF13-R	5'- CTGTATGGTCTTCATTCCGTCA-3'	
OsARF14-F	5'- GGCATATTTATAGAGGCCAGCCT-3'	
OsARF14-R	5'-TCCTTACACCCAATCTTAGTCGG -3'	
OsARF15-F	5'-TCCAGCAAGAGAAGAACTAGA-3'	
OsARF15-R	5'-AGTTGAAATATCAATCACCCT -3'	
OsARF16-F	5'-TTTTTCAGGCCAAAACTTCCTAA-3'	
OsARF16-R	5'-AGGCTCAGACCTGGAAATAATGC -3'	
OsARF17-F	5'-GGCTGGAGCGTCTTTGTAAGT-3'	
OsARF17-R	5'- GGCATAACAGTTTGTGGGCGG-3'	
OsARF18-F	5'-ACCCCATTAGATGGCCTAATTCA-3'	
OsARF18-R	5'- GGCTCACCCTCTTCACATTCTGT -3'	
OsARF19-F	5'- CTTCTGCATAACGTCACTTTAC-3'	
OsARF19-R	5'-TTCTGTCTGTGGTCTCGCTTGT -3'	
OsARF20-F	5'-CTGAGGCTGACGCAGGACT-3'	
OsARF20-R	5'-TCGACCATCTACGCCCATG -3'	
OsARF21-F	5'-CAACCTACGAACCTCTCATCA-3'	
OsARF21-R	5'- TCACAAATTCGGAAGTACTGG-3'	
OsARF22-F	5'-TCACATTCTGCAACAGGTCC-3'	
OsARF22-R	5'- GCTTTTGAGACAGAGGATTC-3'	
OsARF23-F	5'-GAAGGAGTGGATGGTTGTCTA-3'	
OsARF23-R	5'- ACCTCTTCTCTTGTGTAGATG-3'	
OsARF24-F	5'--AGCTCAAGGCTGAGCCTGATAC3'	
OsARF24-R	5'- TCTTTGGACACATTCCCGTTAC-3'	
OsARF25-F	5'-ACAGATGAAGTCTATGCTCAG -3'	
OsARF25-R	5'- GCAGAAGTAATTAGTGGGTTG-3'	
OsSUT1-F	5'-ATCTACCACGGTGACCCAAAG-3'	
OsSUT1-R	5'-GCTGAATCCTAGCACAATCGA-3'	
OsSUT2-F	5'- GTCTGCCTCGTCCTCTTTG-3'	
OsSUT2-R	5'-CCTCTAGTGGCCGCTAATT-3'	
OsSUT3-F	5'-TCATCCCTCAGGTGGTCATCG-3'	
OsSUT3-R	5'-CTTGGAGATCTTGGGCAGCAG-3'	
OsSUT4-F	5'-TTTGGCTGAGCAGAACACCA-3'	
OsSUT4-R	5'-ATGTCATTCGGGCAGAGCTT-3'	
OsSUT5-F	5'-TCGGCATGGTGTCCATGA-3'	
OsSUT5-R	5'-CAATGGCAAGACCTTGGCC-3'	
CPAR2-1-F	5'- CGCAGGTCTCGTTGGTCGCC-3'	ChIP-RT-PCR
CPAR2-1-R	5'-GCATCCGCCTCTCCACTTCCT -3'	

CPAR2-2-F	5'- AGGGCCACCTCGAGCACCTC-3'	
CPAR2-2-R	5'- CGAGAGCAGCAAGCGGCGAG-3'	
CPAR2-3-F	5'-GCTCCCCTCTGCCGCTCTCT -3'	
CPAR2-3-R	5'-AATGGCTGGGCTGGGTGGTG -3'	
CPAR2-4-F	5'- TGACAACCCGCAACCATGCAG-3'	
CPAR2-4-R	5'- TCGACGCTCTAGCTCCCCTCG-3'	
CPAR2-5-F	5'-TGATTAGCCTATGTGATGC -3'	
CPAR2-5-R	5'- ACTTAACTTTAGTCCCTGTATT -3'	
CPAR2-6-F	5'- TGGCAGTTGAGGAGGAGGTA-3'	
CPAR2-6-R	5'-AACGAATGATAGTGAGGTTGTGAC-3'	
ChIP-CH-1-F	5'- CCTCTCTCGCGCGGCTTTCC-3'	
ChIP-CH-1-R	5'- CGCGCCACGCACAAACACAA-3'	
ChIP-CH-2-F	5'- TCTCTCTCCCCCTCTTCCTCCCT-3'	
ChIP-CH-2-R	5'- GGAAATGGAAAGCCGCGCGAGA-3'	
ChIP-CH-3-F	5'- GCTCGGCAAGCGTGTCGCTA-3'	
ChIP-CH-3-R	5'- TGCCAACTGCTATGCTCACCAACA-3'	
ChIP-CH-4-F	5'-CTCCTCCCATCCAGCACG -3'	
ChIP-CH-4-R	5'- GCCAAGAAAGGAAATCGA-3'	
ChIP-CH-5-F	5'- CCAATACTTCGGATCTGTT-3'	
ChIP-CH-5-R	5'- CACTCGACGTTGTAATAGC-3'	
Sequence-OsARF18-1-F	5'- CTTTTGCTCGCTTTGTTCC-3'	Sequence OsARF18 mutant site
Sequence-OsARF18-1-R	5'- GCGTAGCCCTGCTCGTTGG -3'	
Sequence-OsARF18-2-F	5'- TTTCTGGGTTGCGTCTCC-3'	
Sequence-OsARF18-2-R	5'-GGACTGCGTGAGCGTCTT -3'	
Sequence-OsARF2-1-F	5'- TGGGCATCGACCTCAACACC-3'	Sequence OsARF2 mutant site
Sequence-OsARF2-1-R	5'-TCGGCGACCAACGAGACCTG -3'	
Sequence-OsARF2-2-F	5'- CCGCTCCATTAAACCCAA-3'	
Sequence-OsARF2-2-R	5'-CGCCTGCAAACAGCACAA -3'	
Sequence-OsSUT1-F	5'- CTGGGATTCTGGCTTCTTGA -3'	Sequence OsSUT1 mutant site
Sequence-OsSUT1-R	5'- GAAAACAGGGGAAAATGACC-3'	
B-EMSA-OsARF2-1F	5'-CAGGTCTCGTTGGTCGCCGACAACGAGGTGCGTTTGCGTGCGTGCTGATCCAAAAATGGGATGAATATCTTTGACATTC-3'	EMSA-OsARF2-Biotin
EMSA-OsARF2-1F	5'-CAGGTCTCGTTGGTCGCCGACAACGAGGTGCGTTTGCGTGCGTGCTGATCCAAAAATGGGATGAATATCTTTGACATTC-3'	Competitive probe
EMSA-OsARF2-1R	5'-GAATGTCAAAGATATTCATCCCATTTTTGGATCAGCACGCACGCAAACGCACCTCGTTGTCGGCGACCAACGAGACCTG-3'	
B-EMSA-OsARF2-	5'-	EMSA-

1M-F	CAGGTCTCGTTGGTCGCCGACAACGAGGTGCGTTTGCGTGCGTGCTGATCCAAAAATGGGATGAATATCTTTGACATTC -3'	OsARF2-1M-Biotin
EMSA-OsARF2-1M-F	5'-CAGGTCTCGTTGGTCGCCGACAACGAGGTGCGTTTGCGTGCGTGCTGATCCAAAAATGGGATGAATATCTTTGACATTC -3'	Competitive probe
EMSA-OsARF2-1M-R	5'-GAATGTCAAAGATATTCATCCCATTTTTGGATCAGCACGCACGCAAACGCACCTCGTAGACGGCGACCAACGAGACCTG-3'	
B-EMSA-OsARF2-2M-F	5'-CAGGTCTCGTTGGTCGCCGACAACGAGGTGCGTTTGCGTGCGTGCTGATCCAAAAATGGGATGAATATCTTTGTCTTTC -3'	EMSA-OsARF2-2M-Biotin
EMSA-OsARF2-2M-F	5'-CAGGTCTCGTTGGTCGCCGACAACGAGGTGCGTTTGCGTGCGTGCTGATCCAAAAATGGGATGAATATCTTTGTCTTTC -3'	Competitive probe
EMSA-OsARF2-2M-R	5'-GAAAGACAAAGATATTCATCCCATTTTTGGATCAGCACGCACGCAAACGCACCTCGTTGTCGGCGACCAACGAGACCTG-3'	
B-EMSA-ES-1-F	5'-CTCCTCCTCCTACGTCTCCGCCGCTCCTCACTTCCTCCACTCGATTTCCTTTCTTGGCCT- 3'	EMSA-ES1-F-Biotin
EMSA-ES-1-F	5'-CTCCTCCTCCTACGTCTCCGCCGCTCCTCACTTCCTCCACTCGATTTCCTTTCTTGGCCT- 3'	Competitive probe
EMSA-ES-1-R	5'-AGGCCAAGAAAGGAAATCGAGTGGAGGAAGTGAGGAGCGGCGGAGACGTAGGAGGAGGAG -3'	
B-EMSA-ES-2-F	5'-TGCTTCGCCTCTCTCGCTCGTCTCTCCAAACACAAACCCACCACCTCCTCCTCCTCCTCC -3'	EMSA-ES2-F-Biotin
EMSA-ES-2-F	5'-TGCTTCGCCTCTCTCGCTCGTCTCTCCAAACACAAACCCACCACCTCCTCCTCCTCCTCC -3'	Competitive probe
EMSA-ES-2-R	5'-GGAGGAGGAGGAGGAGGTGGTGGGTTTGTGTTTGGAGAGACGAGCGAGAGAGGCGAAGCA-3'	
B-EMSA-ES-3-F	5'-CTCCTCCTCTGACACAGGGGTGTGCAGGTTTGTGTTTGTGCGTGGCGCGTCCGCC-3'	EMSA-ES3-F-Biotin
EMSA-ES-3-F	5'-CTCCTCCTCTGACACAGGGGTGTGCAGGTTTGTGTTTGTGCGTGGCGCGTCCGCC - 3'	Competitive probe
EMSA-ES-3-R	5'-GGCGGACGCGCCACGCACAAACACAAACCTGCACACCCCTGTGTCAGAGGAGGAG -3'	
B-EMSA-ES-4-F	5'-AGAAGCTCGGCAAGCGTGTCGCTAATCCGATACTAACTCC-3'	EMSA-ES4-F-Biotin

EMSA-ES-4-F	5'-AGAAGCTCGGCAAGCGTGTCGCTAATCCGATACTAACTCC-3'	Competitive probe
EMSA-ES-4-R	5'-GGAGTTAGTATCGGATTAGCGACACGCTTGCCGAGCTTCT-3'	
B-EMSA-ES-5-F	5'-TTTGGATAACTACTCGACAGTACAAACGAATTA-3'	EMSA-ES5-F-Biotin
EMSA-ES-5-F	5'-TTTGGATAACTACTCGACAGTACAAACGAATTA-3'	Competitive probe
EMSA-ES-5-R	5'-TAATTCGTTTGTACTGTCGAGTAGTTATCCAAA-3'	
B-EMSA-ES-6-F	5'-ACCGAATTGAATCGAGTCTCCAAAAGAAAAAAGAGAAA -3'	EMSA-ES6-F-Biotin
EMSA-ES-6-F	5'-ACCGAATTGAATCGAGTCTCCAAAAGAAAAAAGAGAAA -3'	Competitive probe
EMSA-ES-6-R	5'-TTTCTCTTTTTTCTTTTGGAGACTCGATTCAATTCGGT - 3'	
B-EMSA-ES-7-F	5'-CAATGTCAAAACCGCGGCATCATATCTCCATTGACGT-3'	EMSA-ES7-F-Biotin
EMSA-ES-7-F	5'-CAATGTCAAAACCGCGGCATCATATCTCCATTGACGT-3'	Competitive probe
EMSA-ES-7-R	5'-ACGTCAATGGAGATATGATGCCGCGGTTTTGACATTG -3'	
B-EMSA-ES-8-F	5'-AAATTGTCACTGTCGCGGTACTGAATTATTCAGACAAT-3'	EMSA-ES8-F-Biotin
EMSA-ES-8-F	5'-AAATTGTCACTGTCGCGGTACTGAATTATTCAGACAAT-3'	Competitive probe
EMSA-ES-8-R	5'-ATTGTCTGAATAATTCAGTACCGCGACAGTGACAATTT -3'	
B-EMSA-ES1-1A-F	5'-CTCCTCCTCCTACATCTCCGCCGCTCCTCACTTCCTCCACTCGATTTCCTTTCTTGGCC - 3'	EMSA-ES1-1A-F-Biotin
EMSA-ES1-1A-F	5'-CTCCTCCTCCTACATCTCCGCCGCTCCTCACTTCCTCCACTCGATTTCCTTTCTTGGCC - 3'	Competitive probe
EMSA-ES1-1A-R	5'-GGCCAAGAAAGGAAATCGAGTGGAGGAAGTGAGGAGCGGCGGAGATGTAGGAGGAGGAG - 3'	
B-EMSA-ES1-2A-F	5'-CTCCTCCTCCTACGACTCCGCCGCTCCTCACTTCCTCCACTCGATTTCCTTTCTTGGCC - 3'	EMSA-ES1-2A-F-Biotin

EMSA-ES1-2A-F	5'-CTCCTCCTCCTACGACTCCGCCGCTCCTCACTTCCTCCACTCGATTTCCTTTCTTGGCC - 3'	Competitive probe
EMSA-ES1-2A-R	5'-GGCCAAGAAAGGAAATCGAGTGGAGGAAGTGAGGAGCGGCGGAGTCGTAGGAGGAGGAG - 3'	
B-EMSA-ES1-3A-F	5'-CTCCTCCTCCTACGTATCCGCCGCTCCTCACTTCCTCCACTCGATTTCCTTTCTTGGCC - 3'	EMSA-ES1-3A-F-Biotin
EMSA-ES1-3A-F	5'-CTCCTCCTCCTACGTATCCGCCGCTCCTCACTTCCTCCACTCGATTTCCTTTCTTGGCC - 3'	Competitive probe
EMSA-ES1-3A-R	5'-GGCCAAGAAAGGAAATCGAGTGGAGGAAGTGAGGAGCGGCGGATACGTAGGAGGAGGAG - 3'	
B-EMSA-ES1-4A-F	5'-CTCCTCCTCCTACGTCACCGCCGCTCCTCACTTCCTCCACTCGATTTCCTTTCTTGGCC - 3'	EMSA-ES1-4A-F-Biotin
EMSA-ES1-4A-F	5'-CTCCTCCTCCTACGTCACCGCCGCTCCTCACTTCCTCCACTCGATTTCCTTTCTTGGCC - 3'	Competitive probe
EMSA-ES1-4A-R	5'-GGCCAAGAAAGGAAATCGAGTGGAGGAAGTGAGGAGCGGCGGTGACGTAGGAGGAGGAG -3'	
B-EMSA-ES1-5A-F	5'-CTCCTCCTCCTACGTCTACGCCGCTCCTCACTTCCTCCACTCGATTTCCTTTCTTGGCC -3'	EMSA-ES1-5A-F-Biotin
EMSA-ES1-5A-F	5'-CTCCTCCTCCTACGTCTACGCCGCTCCTCACTTCCTCCACTCGATTTCCTTTCTTGGCC -3'	Competitive probe
EMSA-ES1-5A-R	5'-GGCCAAGAAAGGAAATCGAGTGGAGGAAGTGAGGAGCGGCGTAGACGTAGGAGGAGGAG -3'	
B-EMSA-ES1-Del-F	5'-CTCCTCCTCCTACCGCCGCTCCTCACTTCCTCCACTCGATTTCCTTTCTTGGCC - 3'	EMSA-ES1-Del-F-Biotin
EMSA-ES1-Del-F	5'-CTCCTCCTCCTACCGCCGCTCCTCACTTCCTCCACTCGATTTCCTTTCTTGGCC - 3'	Competitive probe
EMSA-ES1-Del-R	5'-GGCCAAGAAAGGAAATCGAGTGGAGGAAGTGAGGAGCGGCGGTAGGAGGAGGAG - 3'	
B- EA1-F	5'-GACGGACGAGGTGTACGCGCAGGTCTCGTTGGTCGC- 3'	EA1- F-Biotin
EA1-F	5'-GACGGACGAGGTGTACGCGCAGGTCTCGTTGGTCGC- 3'	Competitive probe
EA1-R	5'-GCGACCAACGAGACCTGCGCGTACACCTCGTCCGTC- 3'	
B-EA2-F	5'-CTCTGCCGCCTACGAGAGACACCCCCACCCCCCC	EA2- F-Biotin

	CCCCCC- 3'	
EA2-F	5'-CTCTGCCGCCTACGAGAGACACCCCCACCCCCCCCCCCCC- 3'	Competitive probe
EA2-R	5'-GGGGGGGGGGGGGTGGGGTGTCTCTCGTAGGCGGCAGAG3'	
B-EA3-F	5'-CACGTGTCATTGGGAGACACGGTTTCAGTCGAGGGGA- 3'	EA3-F-Biotin
EA3-F	5'-CACGTGTCATTGGGAGACACGGTTTCAGTCGAGGGGA- 3'	Competitive probe
EA3-R	5'-TCCCCTCGACTGAAACCGTGTCTCCCAATGACACGTG- 3'	
B-EA4-F	5'-AACTAGGCTTAAAAAAAATGTCTCGTGAATTAGCTTT- 3'	EA4-F-Biotin
EA4-F	5'-AACTAGGCTTAAAAAAAATGTCTCGTGAATTAGCTTT- 3'	Competitive probe
EA4-R	5'-AAAGCTAATTCACGAGACATTTTTTTTAAGCCTAGTT- 3'	
B-EA5-F	5'-ATCTTGGACTAATTCGCGAGACGAATCTATTGAGCCT- 3'	EA5-F-Biotin
EA5-F	5'-ATCTTGGACTAATTCGCGAGACGAATCTATTGAGCCT- 3'	Competitive probe
EA5-R	5'-AGGCTCAATAGATTCGTCTCGCGAATTAGTCCAAGAT- 3'	
B-EA6-F	5'-CACAGTCTCACGGTGGCGAACGCAGCCTTAGACATGAGACGAGAGCA- 3'	EA6-F-Biotin
EA6-F	5'-CACAGTCTCACGGTGGCGAACGCAGCCTTAGACATGAGACGAGAGCA- 3'	Competitive probe
EA6-R	5'-TGCTCTCGTCTCATGTCTAAGGCTGCGTTCGCCACCGTGAGACTGTG- 3'	
B-EA7-F	5'-TCATTTACTCCGCTGGTCTCTCTTCTCCTATTCACTC- 3'	EA7-F-Biotin
EA7-F	5'-TCATTTACTCCGCTGGTCTCTCTTCTCCTATTCACTC- 3'	Competitive probe
EA7-R	5'-GAGTGAATAGGAGAAGAGAGACCAGCGGAGTAAATGA- 3'	
LUC-OsARF2-1-F	5'-	LUC-OsARF2

	TCGACGGTATCGATAAGCTTGAGACGAATCTATTGAGCCT-3'	
LUC-OsARF2-1-R	5'-TAGAACTAGTGGATCCTACCGTAAAAACTACTCCCTACATT-3'	
LUC-OsARF2-2-F	5'-TCGACGGTATCGATAAGCTTAGATCCAAACACCACCTAAGGT-3'	
LUC-OsARF2-2-R	5'-TAGAACTAGTGGATCCTATTGTTGGATAAAGAGAGCGGC-3'	
LUC-OsARF2-3-F	5'-TCGACGGTATCGATAAGCTTCCACCCAGCCCAGCCATTCA-3'	
LUC-OsARF2-3-R	5'-TAGAACTAGTGGATCCGTCCGTCGCGGCGTCCGCCTG-3'	
LUC-OsARF2-4-F	5'-TCGACGGTATCGATAAGCTTCTGCTCCTCCCGTTGTGCT-3'	
LUC-OsARF2-4-R	5'-TAGAACTAGTGGATCCCTCCTGGAATGGCCTCTGC -3'	
LUC-OsSUT1-1-F	5'-TCGACGGTATCGATAAGCTTTTATCCCGTAGCTATTTTACAA-3'	LUC-OsSUT1
LUC-OsSUT1-1-R	5'-TAGAACTAGTGGATCCGGCGGACGCGCCACGCACAAAC-3'	
LUC-OsSUT1-2-F	5'-TCGACGGTATCGATAAGCTTAATCTAAATTCCCCACAAAAC-3'	
LUC-OsSUT1-3-F	5'-TCGACGGTATCGATAAGCTTAGGTCCAATACTTCGGATCTG-3'	
LUC-OsSUT1-4-F	5'-TCGACGGTATCGATAAGCTTTTGAAGTTATTACTGCACTGG -3'	
LUC-OsSUT1-5-F	5'-TCGACGGTATCGATAAGCTTCCCATCCAGCACGCGCCTCCT-3'	
LUC-OsSUT1-6-F	5'-TCGACGGTATCGATAAGCTTTCTCCGCCGCTCCTCACTTCC-3'	
LUC-OsSUT1-7-F	5'-TCGACGGTATCGATAAGCTTCTTGGCCTCTCCTCCTCTGA-3'	

Dataset S1. Functional categories of DEGs in RNA-Seq data identified by Kyoto Encyclopedia of Genes and Genomes (KEGG) analysis.

Dataset S2. GO term analysis of the biological processes enriched in the DEGs of WT and *dao* lodicules.
